# The Socio-Ecological Factors Associated with Mental Health Problems and Resilience in Refugees: A Systematic Scoping Review

**DOI:** 10.1177/15248380241284594

**Published:** 2024-10-08

**Authors:** Tengku Nila Fadhlia, Bertjan Doosje, Disa A. Sauter

**Affiliations:** 1 University of Amsterdam, the Netherlands; 2 Universitas Islam Riau, Pekanbaru, Indonesia

**Keywords:** resilience, mental health, refugees, socio-ecological factors, scoping review, systematic review

## Abstract

Despite the immense challenges to mental health faced by refugees, research consistently finds that many nevertheless demonstrate remarkable resilience. However, a systematic account of the scientific literature on resilience among refugees is currently lacking. This paper aims to fill that gap by comprehensively reviewing research on protective and risk factors affecting refugees’ resilience and mental health problems across four socio-ecological levels: individual, family, community, and society. We conducted a systematic search in the databases PsycINFO, Web of Science, and SocINDEX, as well as contacted topic experts to seek out unpublished manuscripts. This yielded 223 studies (171 quantitative, 52 qualitative), which were subjected to systematic content coding. We found consistent evidence for substantive risk factors, including traumatic experiences and gender at the individual level and postmigration stress and unemployment at the societal level. We found social support to be a clear protective factor at the family and community levels. We discuss these findings in the context of policy and intervention programs and make recommendations at different socio-ecological levels for supporting refugees’ resilience.

## Introduction

Every year in the last decade, the number of people forced to flee has increased because of war, persecution, and human rights violations. There are currently 103 million individuals worldwide who have been forcibly displaced due to force, compulsion, or coercion, including 32.5 million refugees and 4.9 million asylum seekers (United Nations High Commissioner for Refugees [UNHCR], 2022). Refugees meet the eligibility criteria for international protection, and asylum requests have been officially accepted. Asylum seekers are individuals seeking international protection whose claims are still in the process of applying for official refugee status (UNHCR, 2006). Despite the immense adversity inherent to their situation and the high rates of mental health problems in this population, most refugees and asylum seekers show remarkable resilience (Hassan et al., 2016), typically defined as the ability to sustain or improve well-being in conditions of adversity (Ungar, 2018; Ungar & Theron, 2019). The current paper seeks to understand what makes refugees resilient. To address this research question, we map out the risk factors of mental health problems and protective factors of resilience in refugees, organizing them into a general framework for examining factors associated with mental health problems and resilience at four different socio-ecological levels: individual, family, community, and society.

There is a growing research literature investigating refugees’ resilience and mental health problems in relation to a wide range of factors. These include, for example, uncertain asylum status, family separation, language difficulty (Hajak et al., 2021), unemployment, poor housing quality, and perceived discrimination (Farahani et al., 2021). This review aims to provide a general socio-cultural framework into which these empirical findings can be integrated.

There are two primary approaches by which researchers have measured resilience. One is via direct measurement, using scales specifically developed to measure resilience. The other strategy is to measure resilience using related proxy measures, that is, taking mental health problems as an inverse index of resilience. In the current paper, we combine these two approaches to provide a comprehensive synthesis of research on protective and risk factors resilience in refugees.

### Existing Reviews on Refugees’ Resilience

To our knowledge, only two systematic reviews on adult refugees have been conducted to date. One focused more narrowly on three types of mental health problems (anxiety, depression, and posttraumatic stress) as outcomes (Lindert, 2017). The other did not integrate quantitative and qualitative findings (Siriwardhana et al., 2014). Their findings highlighted factors such as family and community cohesion, personal characteristics, collective identity, and religion. However, no framework or grouping was provided for the different factors. Moreover, the research literature has grown considerably since Siriwardhana and colleagues’ systematic review of the field in 2014. Their review was based on 23 articles; we found over 200 relevant articles that had been published since then. Hence, it is time for an update.

There are other reviews of refugees’ resilience, but these focus on specific populations of refugees. These include reviews by Hawkes et al. (2020) and Babatunde-Sowole et al. (2016) who specifically focused on resilience of women with refugee backgrounds. Several reviews have been conducted of young refugees or children (e.g., Pieloch et al., 2016; Polat & Kröner, 2022; Sleijpen et al., 2016); the focus of the current review is on adults, who make up more than half of the refugee population (UNHCR, 2022).

Understanding resilience in refugees is important because resilience is associated with fewer trauma-related mental health problems (Arnetz et al., 2013; Siriwardhana et al., 2014). Poor resilience in refugees predicts psychopathology development (Siriwardhana & Stewart, 2013), while good resilience is associated with better health-related quality of life (Dangmann et al., 2021). The relationship between resilience and mental health indicates that the higher resilience, the lower the mental health problems (Feyissa et al., 2022). Hence, identifying resilience factors is essential to expanding our understanding of how to help refugees attain good mental health.

In the current review, we analyze resilience factors using a socio-ecological framework (Ungar, 2011) consisting of individual, family, community, and societal levels. Such a framework highlights how refugees’ resilience is affected by multiple systems inside and outside of them. By examining risk and protective factors at each socio-ecological level, we aim to provide a holistic framework of resilience in refugees.

### Socio-Ecological Perspective of Resilience in Refugees

Research on resilience faces a significant challenge due to the diverse definitions and conceptualizations of the term (Fletcher & Sarkar, 2013; Ipekci, 2018). Resilience has been variously identified as a trait, a process, or an outcome. Specifically, in the context of forced migration, we propose that resilience is not merely a static trait or an outcome but rather a dynamic process (Ipekci, 2018). Our study, therefore, embraces a dynamic, process-oriented perspective to understand resilience. Following Bronfenbrenner (1979), we take the perspective that multiple systems influence how people deal with forced migration. An individual is the smallest unit in a system impacted by other systems such as family, community, and society. This view is characterized by the interplay of factors across socio-ecological levels—including individual, family, community, and societal influences—each contributing distinctly to how individuals adapt to the challenges of forced migration. We recognize that resilience is subject to change over time and is shaped by continuous interactions between predictors and outcomes.

This dual perspective highlights the complexity of resilience, especially as it is pertinent to refugee experiences. Additionally, Masten (2001) argued for resilience measures that account for risk, protective factors, and outcomes. Our decision to include studies with diverse measurement strategies reflects the multifaceted nature of resilience as an outcome. By examining both protective and risk factors as predictors of resilience, our study acknowledges that resilience is not an inherent trait but is influenced by various external and internal factors. This framework shifts the emphasis of resilience from individual traits to socio-ecological resources and sees individuals as operating within an interconnected network of social relationships (Henderson et al., 2016).

Empirical studies examining resilience in refugees have considered factors at different levels, and the results point to multidimensional factors. However, research to date has not been integrated in a systematic and comprehensive manner. The current review aims to fill that gap by providing a review of the research literature on resilience in adult refugee populations, incorporating mental health problems and resilience factors at four different socio-ecological levels: individual, family, community, and society. Our comprehensive approach provides a deeper understanding of resilience, which is essential for developing adequate support and interventions for refugee populations.

## Methods

### Eligibility Criteria

For this review, we used the following inclusion criteria. We included articles that (a) provided primary data and were peer-reviewed. We also considered unpublished manuscripts that were under review at the time of our article collection. For inclusion, articles also had to (b) be conducted among adult refugees and asylum seekers aged 18 years and over, (c) be written in English, and (d) examine associations, relationships, links, pathways, and causal mechanisms between predictors (protective and/or risk factors) and outcomes indexing resilience in refugees. Resilience outcomes were defined broadly to include both direct measurements of resilience (using a new or established resilience scale, such as the Connor-Davidson Resilience Scale, Brief Resilience Scale, Wagnild & Young Resilience Scale, conceptualized as personal competence, an individual’s ability to “bounce back from stress,” and personal characteristics such as perseverance, and acceptance of self and life) or using a proxy measure of resilience via the measurement of mental health problems. We limited the mental health problems under consideration to depression, anxiety, posttraumatic stress disorder, and psychological distress, as these are the most studied outcomes in the adult refugee population (see Bogic et al., 2015; Siriwardhana et al., 2014).

We thus excluded studies about refugees under 18 years of age, publications in the form of books, book chapters, conference proceedings, reports, magazines, newspaper articles, editorials, reviews, opinions, meta-analyses, commentaries, study protocols, and dissertations. We also excluded studies conducted among refugees from a group perspective (e.g., families of refugees), internally displaced persons, and immigrants other than refugees, as well as research that did not clearly distinguish refugee groups from other possible groups (e.g., ethnic minorities, voluntary migrants). Studies that solely focused on the construction of measures were also excluded.

We acknowledge the importance of measuring promotive factors related to high levels of resilience and positive mental health outcomes. However, the level of competence required to demonstrate resilience can vary based on the severity of an individual’s adversity. For example, for refugees who have experienced severe life adversity, it is appropriate to define competence in terms of the absence of psychiatric diagnoses rather than evidence of excellent functioning or positive mental health outcomes (Fletcher & Sarkar, 2013).

### Literature Search Strategy

We developed a search strategy (available in Supplemental Material 1) in consultation with an academic librarian before searching for papers in three databases: PsycINFO, Web of Science, and SocINDEX, up to June 2022. The search string included variations of the terms *resilience (psychological), mental health, adversity, refugee*, and asylum seeker. We searched the database for research articles from 1806 to 2022, but after applying the eligibility criteria, the search yielded articles dating from 1984 to 2022. In addition to database searches, we also posted on several mailing lists in the social psychology field (i.e., European Association of Social Psychology and Society for Personality and Social Psychology), requesting unpublished under-review manuscripts.

### Article Selection

**Figure 1.**
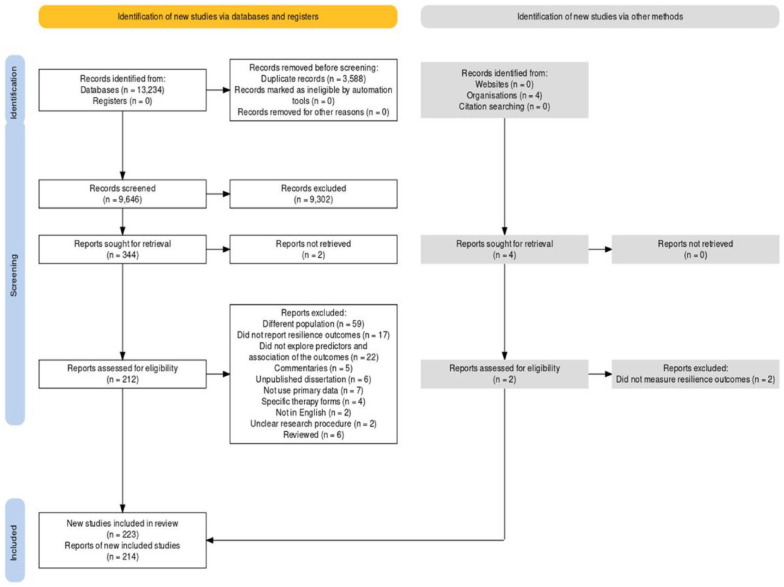
PRISMA 2020 Flow Diagram of Literature Selection.

The first author, with one research assistant, started the selection process by screening for duplicates of all the records identified through the databases and other sources using the reference manager Zotero. Next, we screened the titles and abstracts using Rayyan, a web and mobile application widely used for this purpose (Ouzzani et al., 2016). The inclusion and exclusion criteria were applied during this stage, allowing us to exclude many irrelevant articles based on the eligibility criteria. Next, we retrieved the full text of the remaining articles. For this task, we trained two other research assistants who subsequently acted as two independent reviewers. They conducted the full-text screening and independently decided whether to include or exclude each article. Any disagreements in opinion were discussed and resolved by the third reviewer (the first author). The entire process of literature selection is displayed in Figure 1, which was generated in the *PRISMA*2020 *Shiny app*, a web-based application (Haddaway et al., 2022).

### Data Extraction

We used a semi-structured coding scheme to extract the following information from each study: study characteristics (e.g., publication year, geographic region), study objective, sample characteristics (e.g., sociodemographic data), outcome variables (posttraumatic stress disorder, depression, anxiety, psychological distress, and resilience), outcome measures (mental health problems and direct resilience measures), as well as the risk and protective factors examined. The two reviewers extracted the data independently for each article. First, we tested the coding with three random articles to ensure that the reviewers had the same understanding of the coding scheme (available in Supplemental Material 2). We measured the inter-coder agreement using MAXQDA version 20 (VERBI GmbH, 2020), in which two reviewers process three identical documents independently and code them according to the coding scheme. Any disagreement was discussed until a 100% consensus was reached. Data extraction was also performed using MAXQDA version 20. We coded line by line the factors that were labeled as results or findings in the articles, and after that, the data were transformed into a spreadsheet for further review process.

### Synthesis of Results

We included various study designs in our analysis, which were not homogeneous (Petticrew & Roberts, 2006). Thus, we reported the findings of each type of study in a separate spreadsheet. We conducted individual syntheses for quantitative and qualitative studies, while mixed-method studies were classified as either quantitative or qualitative based on the primary presentation of the findings. Due to the high heterogeneity of the outcome measures across the included studies, we opted for a narrative synthesis for the quantitative results instead of a meta-analysis (Verbeek et al., 2012).

We realize that evaluating the robustness of the evidence is important to make a clear conclusion based on the evidence. Therefore, we conducted a quality appraisal of the quantitative studies. The reason only quantitative studies were assessed was the analysis of the strength and reliability of identified factors derived from quantitative studies that we wrote in our “Discussion” section. Adding the quality appraisal will allow us to conclude this part robustly.

We used a tailored quality appraisal tool that had been used in one systematic review of refugees’ mental health (Bogic et al., 2015). It is a five-point quality appraisal tool devised specifically for their study guided by general guidelines for assessing prevalence studies and key quality criteria identified in previous reviews of refugees’ mental health. The criteria developed as follows:

(1) The sampling method
  (a) The use of random or inclusive sampling (non-random = 0, random or inclusive = 1)
  (b) The sample size if non-random sampling (<200 = 0, ≥200 = 1)
(2) The sampling representativeness, that is, was the sample frame a true or close representation of the target population (not representative = 0, representative = 1)
(3) The response rate (<60% = 0, ≥60% = 1)
(4) The use of validated and reliable measurements (valid and reliable measure not used = 0, valid and reliable measure used = 1)
(5) The interview was conducted in the interviewees’ native language, as opposed to through an interpreter (through interpreter = 0, native language = 1).

The first three criteria relate to the minimization of sample selection bias, while the remaining two relate to the studies’ assessment validity. A cumulative score was calculated for each study. The resulting quality scores ranged from 0 to 5, with lower-quality studies receiving a score of 0 to 3 and high-quality studies receiving a score of 4 to 5 (Bogic et al., 2015).

### Transparency and Openness

We adhered to the PRISMA Extension for Scoping Reviews guidelines for systematic scoping reviews in reporting this review (Tricco et al., 2018). All data and research materials (including our coding scheme) are available at https://osf.io/b79ph/?view_only=86f5db51bdb74e28ba1a29e1aaaa1e71 This review was not preregistered.

## Results

Reports included in the review were published between 1984 and 2022 (see Supplemental Material 3). Out of a total of 214 articles, 162 were quantitative and 52 were qualitative. These contained 171 quantitative and 52 qualitative studies, yielding a total of 223 studies. An overview of the characteristics of the included studies can be found in Table 1.

**Table 1.** Characteristics of Studies Included in the Systematic Scoping Review.

Characteristics of Studies	Quantitative	%	Qualitative	%
Independent samples	171	100	52	100
Region in which studies were conducted				
Europe	49	29	14	27
North America/Canada	58	34	24	46
Africa	8	5	5	10
Australia and New Zealand	19	11	4	8
Middle East	18	11	1	2
South Asia	6	4	2	4
East Asia	10	6	1	2
South America	1	1	—	—
More than one continent	2	1	1	2
Sample size				
<10	—	—	18	35
11–50	12	7	29	56
51–100	34	20	5	10
101–200	32	19	—	—
201–500	56	33	—	—
>500	37	22	—	—
Origin of samples				
Middle East	51	30	9	17
Africa	22	13	16	31
East Asia	13	8	1	2
South Asia	42	25	10	19
The Balkans	12	7	1	2
Central America	1	1	1	2
The Caribbean	—	—	1	2
Refugees from mixed countries of origin	30	18	13	25
Sample selection				
Random	26	15	—	—
Convenience	94	55	15	29
Purposive	8	5	20	38
Snowball	10	6	8	15
Mixed (purposive, snowball, convenience)	17	10	8	15
Other	16	9	1	2

*Note*. Raw numbers and percent shown separately for quantitative and qualitative studies for region of study, sample size, region of origin of refugees, and sample selection.

The quantitative studies in our review were mainly cross-sectional (152 studies), with only a small number of longitudinal (17 studies) and intervention studies (2 studies). We excluded intervention studies that aimed to test specific therapies, as their results could not be integrated with the overall review. The quantitative studies employed various measurements to assess mental health outcomes: the Hopkins Symptom Checklist-25 was used in 49 studies to measure depression and anxiety, and the Harvard Trauma Questionnaire was used in 40 studies to measure posttraumatic stress. Qualitative studies, on the other hand, primarily explored resilience directly through in-depth interviews.

Due to space limitations, it is not feasible to provide a summary of each study here. However, we provide a summary of each study, detailing dependent and independent variables, socio-ecological levels examined, the purpose of the study, sample characteristics, protective and risk factors probed, and outcome variables in Supplemental Materials 4 and 5. Below, we present the main findings in terms of the protective and risk factors examined in relation to refugees’ resilience, organized within the socio-ecological framework structure (individual, family, community, and society). This review integrates the results from both quantitative and qualitative research by first summarizing the main findings from each and then synthesizing them across quantitative and qualitative studies.

### Summary of the Main Findings from Quantitative and Qualitative Studies

#### Quantitative Studies

We extracted 26 factors related to resilience from the quantitative studies. Results varied considerably in terms of consistency; we only included factors for which associations with resilience were reported as statistically significant (*p* < .05) in more than three studies (Bogic et al., 2015). The consistency of each factor can be seen in Supplemental Material 3. A summary of the quantitative findings can be seen in Supplemental Material 3.

#### Qualitative Studies

We identified 9 risk factors and 14 protective factors from the qualitative studies. Following the same structure as the quantitative studies, we organized them into the socio-ecological framework, as shown in Supplemental Material 3.

### Risk and Protective Factors

In this section, we synthesize resilience factors at each level of the socio-ecological structure. Table 2 displays the summary of factors contributing to refugees’ resilience at each socio-ecological level. We modified the socio-ecological model by Babatunde-Sowole et al. (2016) and assigned the factors to each level, referring to previous reviews that adopted this framework. At the individual level, we place factors such as thoughts, behaviors, personal values, and skills (Babatunde-Sowole et al., 2016). As for the societal level, the factors related to programs, assessments, and policies from the host country affect the lives of refugees (Farahani et al., 2021). In their review, Scharpf et al. (2021) place postmigration stress, acculturation, resettlement location, ethnic origin, and placement type on the societal level. We argue that refugees’ unemployment results from the host country’s economic and political environment. Thus, we place it on a societal level, too. We describe risk factors as predictors of higher levels of mental health problems and protective factors as predictors of lower levels of mental health problems. For each factor, we integrate results from both quantitative and qualitative studies.

**Table 2.** Summary Key of Findings.

The Socio-Ecological Resilience Factors in Refugees	Findings
Individual-level risk factors	Studies overwhelmingly indicate a positive association between traumatic experiences and various mental health problems.
	On average, females report more mental health problems than males, but in over one-third of studies, no relationship between gender and mental health issues was found.
	Age is not associated with resilience in most studies, but in one-third of studies, age is linked to worse mental health outcomes.
	Lower socio-economic status predicts worse mental health problems and lower resilience, and both physical and mental health problems in the past are clearly associated with greater risk of current mental health issues.
	There is also evidence that maladaptive coping strategies and language difficulties are correlated with worse mental health outcomes.
Individual-level protective factors	There is mixed evidence indicating a possible positive relationship between higher education and better resilience.
	There is evidence from both quantitative and qualitative studies suggesting that positive coping and religiosity constitute protective factors.
	More tentative evidence point to spirituality, positive emotional experience, personality traits, self-agency, and self-efficacy as protective factors for resilience.
Family-level risk factors	Poor living conditions have consistently been found to map to worse mental health problems.
	Financial strain is also a risk factor for low resilience, as are physical complaints and traumas experienced by loved ones, though relatively few studies have investigated these factors.
Family-level protective factors	Both quantitative and qualitative studies provide consistent evidence that family support is a protective factor for refugees’ resilience.
	Many studies have examined the relationship between marital status and mental health problems; though most evidence suggests a null relationship, one-third of studies indicate that being married is related to better mental health.
	In qualitative studies, a family-derived sense of purpose gives meaning to attaining a better life, and promotes resilience.
Community-level risk factors	Findings from a few quantitative studies suggest that social isolation is associated with worse mental health problems in refugees.
	Qualitative studies have highlighted the negative effect that refugees’ ethnic communities can have on their resilience.
	It is worth noting that community-level factors have been less investigated than the other socio-ecological levels in the context of refugees’ resilience.
Community-level protective factors	Quantitative and qualitative studies provide evidence that community support and social support constitute protective factors.
	Despite somewhat inconsistent results, most quantitative evidence suggests that a strong cultural identity is a protective factor, and the qualitative findings support this conclusion.
Societal-level risk factors	Quantitative and qualitative studies have consistently found a positive association between postmigration stress and mental health problems.
	Most quantitative studies have found a positive association between perceived discrimination and mental health problems.
	Unemployment is also consistently linked to a higher prevalence of mental health problems in refugees.
	There is some evidence from qualitative studies that longer stays in refugee camps are associated with a higher prevalence of mental health problems.
	Despite no qualitative evidence, longer stays in a reception country and lack of legal status have been linked to mental health issues and lower resilience in quantitative studies.
	Qualitative findings suggest that lack of professional recognition is a significant risk factor, but no evidence on this factor exists from quantitative factors.
Societal-level protective factors	Evidence indicates that integration or acculturation to the host culture is associated with fewer mental health problems and is essential for refugees to develop their resilience.
	A sense of safety is identified as something society offers, which forms the basis of opportunities.
	Access to opportunities is related to resources from a society that refugees can benefit from; it has been positively associated with resilience in qualitative studies.

#### Individual-Level Factors

##### Risk Factors

At the individual level, a considerable number of risk factors have been established. These relate to demographics (age, gender, and socio-economic status [SES]), traumatic experiences, previous health and mental health problems, maladaptive coping, and language difficulties. We discuss the findings per factor in turn, ordered by how frequently they have been studied.

###### Traumatic Experiences

More than half of the quantitative studies (*n* = 100) tested the association between traumatic experiences and mental health problems. The findings are remarkably consistent, with 98 studies (98%) finding that traumatic experiences are linked to higher rates of mental health problems, including PTSD (post-traumatic stress disorder) (e.g., Acarturk et al., 2018; Vromans et al., 2020), depression (e.g., Sangalang et al., 2019; Starck et al., 2020), anxiety (e.g., Birman & Tran, 2008; Gerritsen et al., 2006), and psychological distress (e.g., Alemi & Stempel, 2018; Stempel et al., 2016).

Aligning with these results, traumatic experiences were also identified as a risk factor in 15 qualitative studies. The experience of war-related trauma before coming to the host country affects the ability to face daily challenges (Kuttikat et al., 2018) and trauma from the past (Yotebieng et al., 2019), separation from family (Thomas et al., 2011), famine, and human rights abuses (Han et al., 2020) have all been found to impair refugees’ resilience.

###### Gender, Age, and SES

Findings on age, gender, and SES only come from quantitative studies; no evidence on these factors is available from qualitative studies. The results for gender point to being female being associated with a higher risk of mental health problems, a pattern reported in 48% out of 89 studies that included gender as a factor. Being female has been found to be associated with more significant PTSD symptoms (e.g., Kaya et al., 2019; Riley et al., 2017), and women report a higher prevalence of depressive and anxiety symptoms (e.g., Acarturk et al., 2021; Sengoelge et al., 2020). However, 34 studies (38%) of studies that have examined the relationship between gender and mental health problems have found no significant relationship (e.g., Chung et al., 2018; Kartal & Kiropoulos, 2016). In sum, these results indicate that, on average, females experience more mental health problems than males.

In 45 (56%) out of 80 studies, no correlation between age and mental health problems was found (e.g., Acarturk et al., 2018, 2021; Hamrah et al., 2020a). However, in 28 studies (35%), researchers found a significant positive correlation between age and mental health problems. For example, increased age is associated with higher psychological distress (e.g., Alemi & Stempel, 2018; Kim, Yun, et al., 2019). Stronger symptoms of PTSD are also significantly associated with age (Georgiadou et al., 2018), such that a higher rate of PTSD is more common in older than in younger age (Ahmad, Othman, & Lou, 2020; Cheung, 1994).

SES has only been examined in 15 studies, but out of those, 9 studies (60%) have found that low SES is linked to more pronounced mental health problems. For example, refugees with lower incomes are more likely to be depressed than refugees with higher incomes (e.g., Cummings et al., 2011; Nicholson, 1997), and poverty is linked to higher symptoms of anxiety (Acarturk et al., 2021; Silove et al., 1997), PTSD (Beiser et al., 2011), and psychological distress (Saab et al., 2020). Evidence to date suggests that lower SES is positively associated with mental health problems and, thus, lower resilience.

###### Chronic Physical and Mental Health Issues

For chronic physical health issues, all 18 studies (100%) that have investigated this factor have found that having previous physical health problems positively correlates with the risk of having current mental health problems. Physical health problems that have been examined include neurological disorders (Naal et al., 2021), somatic complaints (Bentley et al., 2011), disability (Acarturk et al., 2021; Hossain et al., 2020), and general physical illness (e.g., Kaya et al., 2019; Taylor et al., 2014). Four qualitative studies have identified chronic physical health issues as a risk factor (e.g., Denzongpa & Nichols, 2020). Refugees face challenges in trying to cope with chronic disease as newcomers in a host country (Miller, Worthington, et al., 2002; Yotebieng et al., 2019), often with limited access to the health care system in the new country (Goodman et al., 2017).

In 18 quantitative studies (95%) out of 19, researchers report that having a previous history of mental health issues is associated with a higher risk of having current mental health problems. For example, 11 studies found that psychiatric disorder in the past was a predictor of PTSD (e.g., Javanbakht et al., 2020). Four longitudinal studies also indicate that the most powerful predictor of later depression is baseline depression scores (e.g., Beiser et al., 1993; Hinton et al., 1997).

###### Maladaptive Coping

Coping has been examined as a predictor of refugees’ resilience in 20 quantitative studies. Maladaptive (sometimes referred to as negative) coping refers to the use of coping strategies or behaviors that do not effectively address a stressful situation and may even create additional problems (e.g., denial, self-blame, self-distraction, and substance use) (Cooper et al., 2006). In 8 (40%) out of 20 studies, the use of maladaptive coping was found to be associated with higher scores of depression (e.g., Vonnahme et al., 2015; Woltin et al., 2018) and PTSD (Demir et al., 2020; Nickerson et al., 2011). Maladaptive coping has not been identified as a risk factor for resilience in qualitative studies. Thus, less than half of the quantitative studies suggest that maladaptive coping is associated with worse mental health problems, while the rest of the studies show that positive coping is negatively associated with mental health issues, which we will explain in the “Protective Factors” section.

###### Language Difficulty

Language has been examined in 27 quantitative studies, with 19 studies (70%) indicating that language difficulty is associated with worse mental health outcomes. For example, low proficiency in English is significantly correlated with depression (e.g., Beiser & Hou, 2006; Cummings et al., 2011), and difficulty communicating in English has been found to be associated with higher rates of PTSD (Hamrah et al., 2020a; Tinghög et al., 2017), psychological distress (Alemi et al., 2015), and anxiety (Chung & Kagawa-Singer, 1993).

Findings from qualitative studies corroborate the results from quantitative research in this domain. A primary challenge of settling in a new country was reported by refugees to be learning a new language (Lenette et al., 2013; Miller, Worthington, et al., 2002). Language and communication barriers might hinder refugees’ possibilities to navigate their new society, including education and work systems (e.g., Ganassin & Young, 2020; Hasan et al., 2018).

##### Protective Factors

At the individual level, the research literature has identified several protective factors, including higher education, positive coping, religiosity, positive emotional experiences, personality traits, self-agency, spirituality, and self-efficacy. Interestingly, many protective factors have only been identified in qualitative studies.

###### Higher Education

Education has been investigated in 52 quantitative studies, of which 31 studies (60%) found no correlation between education and resilience. However, 21 studies (40%) did report a significant association. Specifically, in 18 studies (35%), researchers found a significant *negative* correlation between the level of education and mental health problems. For example, studies found that refugees or asylum seekers with higher education reported fewer depression symptoms (e.g., Smeekes et al., 2017; Ying et al., 1997), lower rates of PTSD (e.g., Carlsson et al., 2006; Hussain & Bhushan, 2011), and lower rates of psychological distress (Alemi & Stempel, 2018; Alemi et al., 2015). To conclude, most of the evidence points to a null relationship between education and resilience, but one-third of studies have found higher education to be a protective factor.

###### Positive Coping

Of the 20 quantitative studies that investigated coping, 6 studies (30%) showed that positive coping, either problem-focused or emotion-focused, was a protective factor for mental health problems. Engaging in coping is associated with less severe symptoms of PTSD (Lindencrona et al., 2008; Poudel-Tandukar et al., 2020), and depression (Miller, Weine, et al., 2002; Noh et al., 1999). A smaller number of studies (*n* = 4, 20%) have found no association between coping and mental health problems (e.g., Ahmad, Othman, Hynie, et al., 2020; Lim & Han, 2016).

In 23 qualitative studies, participants have reported different types of coping strategies to aid their resilience. Emotion-focused coping (directed at regulating emotional response to the problem) was found to be utilized more than problem-focused coping (directed at managing the problem or altering the problem causing the distress) by participants. One of the strategies employed was cognitive reframing, which altered the way they looked at their past (Goodman et al., 2017; Pineteh, 2017; Tippens, 2017), and helped them create an internal narrative (Lavie-Ajayi & Slonim-Nevo, 2016). This allowed them to make sense of the difficulties they had faced and to accept their present life, thus helping them become resilient.

###### Religiosity

Religiosity has been examined in eight quantitative studies, with five studies (63%) finding it to constitute a protective factor. Three studies showed that faith was associated with lower rates of PTSD (Bentley et al., 2014; Denkinger et al., 2021; Mölsä et al., 2017), and similar results have been found for depression symptoms (Kuittinen et al., 2017). Religiousness has also been linked to a lower likelihood of psychological distress (Tippens et al., 2021).

In line with this result, most qualitative studies (30) noted religiosity as one of the essential factors promoting resilience. Faith in God helped refugees cope in challenging times (e.g., Corley & Sabri, 2020; Maung et al., 2021). Religious refugees felt grateful to God for overcoming their difficulties and traumas and surviving adversities (Abraham et al., 2018), and also gave them hope for the future (Babatunde-Sowole et al., 2020; Hasan et al., 2018).

###### Positive Emotional Experiences

Positive emotional experiences emerged as a resilience factor in 17 qualitative studies, but this factor has yet to receive attention in quantitative studies. Despite the hardships they had faced, refugees and asylum seekers still managed to feel gratitude (Lenette et al., 2013; Verreault, 2017; Welsh & Brodsky, 2010). In several studies, participants talked about hope for the future (Kuttikat et al., 2018; Lavie-Ajayi & Slonim-Nevo, 2016) and maintaining a positive outlook (Aube et al., 2019; Ganassin & Young, 2020) to give them strength.

###### Personality Traits

Personality traits are another protective factor that has only been examined in qualitative studies. Personality traits that allowed refugees to endure highly adverse life circumstances have been described in 14 studies. These include strong self-determination (Maung et al., 2021), persistence (Lusk et al., 2019), a vision for the future (Melamed et al., 2019), proactivity (Liu et al., 2020), creativity (Lenette et al., 2013), flexibility (Hormozi et al., 2018), independence (Denzongpa & Nichols, 2020), and ingenuity (Babatunde-Sowole et al., 2020). Given that these studies have examined a wide range of non-overlapping personality traits, evidence for this factor should still be considered preliminary.

###### Self-Agency

Self-agency was mentioned as a protective factor in eight qualitative studies, and evidence on this factor is thus limited. Studies point to a sense of being capable (e.g., Simich et al., 2010; Yotebieng et al., 2019) and a sense of mastery (Verreault, 2017) as a way to build self-agency. One study of single refugee women found that those who maintained a strong focus on their goals achieved resilience irrespective of support (Lenette et al., 2013).

###### Self-Efficacy

Self-efficacy has been investigated in seven quantitative studies, of which four (57%) found a positive association with resilience. No evidence from qualitative studies is available concerning this factor. Two studies conducted among Syrian and North Korean refugees both found that self-efficacy was significantly correlated with resilience (Lim & Han, 2016; Pak et al., 2022). Two other studies found that self-efficacy was associated with lower rates of PTSD (Chung & Shakra, 2020; Von Haumeder et al., 2019). Thus, a small number of quantitative studies point to self-efficacy as a protective factor, though a considerable proportion (43%) of the studies has found no correlation.

###### Spirituality

Spirituality has been identified as a source of resilience separate from religiosity in five qualitative studies (Pearce et al., 2016; Verreault, 2017). For example, faith in a higher power is a source of strength and optimism (Kuttikat et al., 2018). In another study, natural imagery was often described as spiritually uplifting (Munt, 2012).

#### Family-Level Factors

##### Risk Factors

There are important risk factors for refugees’ resilience that occur at the family level. These include poor living conditions, financial strain, and health problems or trauma of loved ones.

###### Living Conditions

Nine quantitative studies have tested the association between living conditions and mental health risks. Findings show that poor satisfaction with living conditions, not living with a partner, living alone, and small family size in the host country are associated with high rates of depression (e.g., Ahmad, Othman, Hynie, et al., 2020; Feyera et al., 2015), PTSD (Sengoelge et al., 2019), and psychological distress (Walther, Fuchs, et al., 2020). Thus, evidence is consistent but based on a relatively small number of quantitative studies and suggests that unsatisfactory living conditions are associated with worse health outcomes.

###### Financial Strain

Evidence from five quantitative studies shows that refugees who consider themselves providers for their families but are unable to pay the monthly bills are highly likely to report depression (Sengoelge et al., 2020; Vonnahme et al., 2015), and psychological distress (Alemi et al., 2015). Findings from qualitative studies resonate with this. Ten qualitative studies described that the majority of refugees sampled faced severe financial strain, which negatively affected their resilience (e.g., Abur & Mphande, 2020; Tippens, 2017). To summarize, consistent evidence indicates that financial strain is a risk factor for resilience at the family level, though findings are based on a small number of studies.

###### Health Problems or Trauma Experienced by Loved Ones

Only four quantitative studies have investigated health problems or trauma experienced by loved ones as a risk factor for resilience. Findings consistently point to physical complaints and traumas experienced by loved ones being associated with poorer mental health outcomes. For example, a family member having suffered from a psychiatric disorder or a loved one having been tortured increased the likelihood of depression and PTSD (e.g., Acarturk et al., 2018; Cummings et al., 2011).

##### Protective Factors

At the family level, research has identified three protective factors: family support, marital status, and family-derived sense of purpose. We placed a sense of purpose at the family level because the family is described as the source of purpose in these studies.

###### Family Support

Social support can be derived from multiple sources, and family is one of them. There is clear evidence that social support is associated with mental health, with 24 quantitative studies (80%) out of 30 finding that social support functions as a protective factor. A support system (i.e., family) is associated with a less frequent diagnosis of PTSD (Tufan et al., 2013) and depression (Birman & Tran, 2008). One study with Arabic-speaking refugees in Berlin and Amman found that perceived social support positively influenced their mental health: In the Berlin participants, the support offered by a significant was associated with fewer depressive symptoms, while family support decreased psychological symptoms in the Amman sample (Böge et al., 2020). However, three studies (16%) have not found a significant relationship between social support and psychological problems (Ai et al., 2002; Böge et al., 2020; Hooberman et al., 2010).

In line with these quantitative findings, social support from family is a resilience factor identified in 16 qualitative studies. Social support emerged as a contributor to overcoming mental health issues (Han et al., 2020). Sources of social support might be varied but typically include family members. Turning to the family for emotional support was perceived as comforting during difficult times, creating a sense of strength (Corley & Sabri, 2020; Davis, 2000).

###### Marital Status (Being Married)

The relationship between marital status and mental health problems has been examined in 34 quantitative studies. Of these, 50% found no significant link (e.g., Alduraidi et al., 2020; Arnetz et al., 2013; Kahve et al., 2019). However, one-third of the studies (35%) found a negative association between being married and mental health problems, thus pointing to being married being a protective factor for resilience. Three studies identified marriage as a protective factor for developing psychological problems (Beiser et al., 1993; Beiser & Hou, 2006; Nosè et al., 2018). One study reported that the likelihood of having depressive symptoms was higher in people who were divorced (Feyera et al., 2015). One study reported that being single, divorced, or widowed was associated with increased psychological distress (Walther, Fuchs, et al., 2020). Two studies reported that being single was a significant predictor of depression (Nesterko et al., 2020) and PTSD (Hussain & Bhushan, 2011). There is no evidence from qualitative studies concerning this factor.

###### Family-Derived Sense of Purpose

A family-derived sense of purpose was found in 10 qualitative studies be a source of resilience. Primarily, children gave a sense of purpose to refugees that motivated them to embark on the perilous migration journey (Aube et al., 2019; Lusk et al., 2019). They found the courage to undertake the journey in order to give their children a safe space to grow up (Lusk et al., 2019). Refugees put the well-being of their children first and want their children to benefit from new opportunities (Lenette et al., 2013). Parenthood appears to, in some cases, counteract everyday psychological challenges (Thomas et al., 2011).

#### Community-Level Factors

##### Risk Factors

The *community level* is the level operating outside of the more immediate family level but on a smaller and more local scale than society at large. At the community level, we identified two risk factors relating to refugees’ ethnic community: social isolation and community scrutiny.

###### Social Isolation

Two quantitative studies have shown that a deficit in social connection increases the likelihood of depression and anxiety (Gautam et al., 2021; Hamrah et al., 2020a). Two further studies have found that social isolation is associated with more PTSD symptoms (Miller, Weine, et al., 2002; Morgan et al., 2017). Thus, findings from a small number of quantitative studies suggest that social isolation is associated with worse mental health problems in refugees.

###### Community Scrutiny

Besides being a protective factor, the community can also be a risk factor due to the unsolicited high level of scrutiny they can give to their members. Seven qualitative studies have highlighted the negative effects that refugees’ ethnic communities can have on resilience. For example, unhealthy competition and gossip within the community can harm resilience (Rahapsari & Hill, 2019). The community can be a source of stress because of scrutiny and gossip, especially when targeting single refugee women who raise children without a spouse (Lenette et al., 2013). Furthermore, refugees can also face scrutiny from the community for disclosing their mental health problems because of the stigma attached (Corley & Sabri, 2020). Thus, these studies outline that ethnic communities can negatively relate to refugees’ resilience due to high levels of perceived scrutiny, gossip, and unhealthy competition.

##### Protective Factors

At the community level, we found community support (social support that takes place at the community level), positive social relations, and cultural identity to constitute protective factors. Although not every study that measured social support differentiated the support source, some indicated the assessment tools they used, allowing us to utilize their findings.

###### Community Support

In 24 quantitative studies (80%) out of 30, a negative association was found between social support and mental health. For example, refugees who perceive strong social support from their social network or friends in daily life have lower odds of depression (Birman & Tran, 2008; Vonnahme et al., 2015). Similarly, having a support system from fellow villagers is associated with lower rates of PTSD (Tufan et al., 2013).

Sixteen qualitative studies also indicate a role for social support from the community. When facing difficult circumstances, social support can be sought from different sources, including friends and online communities (Udwan et al., 2020) and can include emotional as well as practical support. In a study with Congolese refugees in Kenya, refugees established a borrowing network within the Congolese community to provide economic support (Tippens, 2017). Refugees rely on other community members for help and support (Kuttikat et al., 2018), which can help reduce stress (Corley & Sabri, 2020).

###### Cultural Identity

Ethnic identity was examined in 14 quantitative studies, and 6 (43%) found that it constitutes a protective factor. A high level of ethnic identity has been found to buffer the psychologically detrimental effect of the resettlement stressors (Beiser & Hou, 2006). Several studies have found that stronger identification with one’s identity of origin is related to lower levels of depression (Çelebi et al., 2017) and psychological distress (Walther, Fuchs, et al., 2020). In contrast with the previous findings, five studies found that strong intra-ethnic identity was positively associated with higher levels of psychological distress (e.g., Alemi & Stempel, 2018; Birman & Tran, 2008). Lastly, no relationship was found between ethnic identity and mental health in three studies (Alemi et al., 2015; Noh et al., 1999; Starck et al., 2020). Thus, although there are somewhat inconsistent results, more evidence points to ethnic identity as a protective factor.

Qualitative results resonate with the quantitative findings, with cultural identity contributing to refugees’ resilience. For example, some refugees feel that dance and other cultural elements from their home country help to sustain their resilience (Munt, 2012; Verreault, 2017). Connecting to others from their own cultural background (Lenette et al., 2013), practicing one’s own culture freely (Liu et al., 2020), and attending cultural events can bolster the feeling of belonging (Kuttikat et al., 2018).

###### Social Relations

Six quantitative studies have examined social relations, and all found a positive association with resilience. Here, we frame social relations as interactions with social network members, specifically community group members. In this context, it is both about the quality and quantity of the network. For example, a study with North Korean refugees residing in South Korea showed that participants with positive social relationships were less likely to exhibit depressive symptoms (Um et al., 2015), and a study with Syrian refugees found a negative correlation between social relations and PTSD symptoms (Pak et al., 2022).

Findings from qualitative studies corroborate these quantitative results. Being a part of a group is important for refugees’ resilience (Verreault, 2017). Importantly, social relations are established online as well as in person. A qualitative study in the Netherlands found that social media connections between Syrian refugees functioned as a digital resilience resource because it allowed them to feel connected and close (Udwan et al., 2020). Community affiliations (Babatunde-Sowole et al., 2020; Davis, 2000) have been deemed beneficial for refugees to develop resilience.

#### Societal-Level Factors

##### Risk Factors

In this part, we describe societal-level factors, which include risk factors from broader society in the host country. It includes postmigration stress, unemployment, perceived discrimination, non-permanent legal status, lack of professional recognition, length of stay in refugee camps, and length of stay in the host country. These factors can have a significant impact on the mental health and resilience of refugees and are often particularly influenced by the policies and practices of the host country.

###### Postmigration Stress

Most of the studies defined postmigration stress as the stress in relation to postmigration stressors, such as current culture-related stress (Jeon et al., 2013), resettlement stress (Frounfelker et al., 2022), and stress arising from postmigration living difficulties (Steel et al., 2011). Thirty-six quantitative studies have tested postmigration stress as a predictor, and all found that it correlates positively with mental health problems. Postmigration stress (e.g., Nakash et al., 2015; Sangalang et al., 2019), as well as general postmigration problems (e.g., Silove et al., 1998; Vromans et al., 2020), are correlated with mental health problems among refugees.

This pattern of results is consistent with findings from qualitative studies. For refugees, adjusting to a country with a different culture is challenging, with many experiencing cultural shock (Aube et al., 2019). Many experience difficulties in adjusting to the majority of cultural norms and practices (Abur & Mphande, 2020; Groen et al., 2019; Lenette et al., 2013) and living as an ethnic and religious minority member (Hasan et al., 2018; Hormozi et al., 2018). Overall, the evidence from quantitative studies indicates a positive association between postmigration stress and mental health problems, and qualitative studies also show that experiencing postmigration challenges can lead to psychological problems and, therefore, undermine resilience.

###### Unemployment

Unemployment was investigated in 30 quantitative studies, of which 18 (60%) reported a positive association between unemployment and the prevalence of mental health problems, including PTSD (e.g., Ahmad, Othman, & Lou, 2020; Teodorescu et al., 2012), depression (Beiser et al., 1993; Beiser & Hou, 2006), and psychological distress (Alemi et al., 2015; Walther, Kröger, et al., 2020). In contrast, 11 studies (37%) found no correlation between unemployment and PTSD, depression, or psychological distress (e.g., Arnetz et al., 2013; Kashyap et al., 2019). There are 13 qualitative studies that have found that unemployment is a source of mental health problems in refugees (e.g., Akinyemi et al., 2016; Thomas et al., 2011).

###### Perceived Discrimination

Perceived discrimination has been investigated in 15 quantitative studies, with 14 (93%) showing a positive correlation with the likelihood of mental health problems. Perceived discrimination has been found to be significantly associated with worse depression (e.g., Beiser & Hou, 2006; Borho et al., 2020), PTSD symptoms (e.g., Mölsä, Tinghög, et al., 2017), anxiety (Sangalang et al., 2019), and psychological distress (Alemi & Stempel, 2018; Stempel et al., 2016).

Findings from 15 qualitative studies also point to a relationship between refugees’ resilience and their perception of discrimination. Refugees frequently report perceiving that they have been discriminated against, and discrimination is experienced as a major source of mental health issues after resettlement (Melamed et al., 2019). Refugees report feeling discriminated against based on their ethnicity or religion (Goodman et al., 2017), as due to language (Babatunde-Sowole et al., 2020) and appearance (e.g., head scarf). Refugees reported experiencing discrimination in the process of finding a place to live (Aube et al., 2019), economic transactions (Babatunde-Sowole et al., 2020), and employment (Akinyemi et al., 2016; Goodman et al., 2017).

###### Non-Permanent Legal Status

Legal status refers to recognition provided by governments to forced migrants, and specifics depend on the laws and policies of that country (Lewis & Rödlach, 2019). Legal status has been examined in 13 quantitative studies, of which 12 (92%) found that non-permanent legal status was associated with worse mental health problems. For example, holders of temporary visas or shorter validity of residence permits reported more significant PTSD symptoms (e.g., Borho et al., 2020; Georgiadou et al., 2018), depression (Morgan et al., 2017), anxiety (Gerritsen et al., 2006), and psychological distress (Walther, Kröger, et al., 2020) than holders of longer or permanent residence permits. There is no evidence concerning this factor from qualitative studies.

###### Lack of Professional Recognition

In seven qualitative studies, lack of recognition of refugees’ previous qualifications was found to have a negative association with resilience. Many refugees have difficulty finding a job that meets their skills and qualifications because the work system in the host country does not recognize their previous education and qualifications (e.g., Goodman et al., 2017; Schweitzer et al., 2007; Stempel et al., 2016). Lack of recognition of previous qualifications frequently results in unemployment or underemployment, affecting refugees’ mental health negatively (Akinyemi et al., 2016). It is worth noting that evidence on this factor comes only from qualitative findings.

###### Length of Stay in Refugee Camps

Seven quantitative studies consistently indicate that longer stays in refugee camps are associated with higher rates of mental health problems. For example, a longer stay in a detention camp was associated with higher PTSD symptoms (e.g., Keller et al., 2003; Z. Steel et al., 2006), as well as more anxiety and depression (Chung & Kagawa-Singer, 1993). One study also found that dissatisfaction with the refugee camp conditions increased depression (Acarturk et al., 2018). No qualitative data is available on this factor.

###### Length of Stay in Host Country

Nine quantitative studies have examined whether the length of stay in the host country predicts mental health problems. Six studies (67%) found that the longer the stay in the host country, the worse the refugees’ mental health problems. Three studies found that refugees who had lived relatively long in the United States and Canada had higher rates of depression (Ahmad, Othman, Hynie, et al., 2020; Chung & Kagawa-Singer, 1993; Taylor et al., 2014). Two studies have shown the opposite relation: a longer time in the host country is associated with *decreased* distress (Nosè et al., 2018; Walther, Fuchs, et al., 2020). No evidence of this factor exists from qualitative studies.

##### Protective Factors

At the societal level, three protective factors have been identified: acculturation/integration, access to opportunities, and a sense of safety. These factors occur at a broader societal and cultural level in the host country.

###### Integration/Acculturation

Eleven quantitative studies tested integration or acculturation to the host culture, and nine of them (82%) reported a negative relationship with the prevalence of mental health problems. This suggests that greater integration/acculturation is associated with better mental health outcomes. Sociocultural adaptation (Um et al., 2015) and orientation toward the host culture (Jorgenson & Nilsson, 2021; Starck et al., 2020) are associated with lower rates of depression. Participation in integration courses is associated with less psychological distress (Walther, Kröger, et al., 2020), as is spending more time with members of the host culture (Walther, Fuchs, et al., 2020). Only two studies have reported no relationship between the two variables (Mölsä et al., 2017; Nakash et al., 2015).

There is also evidence of this relationship from 13 qualitative studies. Contributing to the host society can function as a way of finding a new sense of belonging (Ganassin & Young, 2020), redefining identity (Lavie-Ajayi & Slonim-Nevo, 2016), and overcoming depression (Denzongpa & Nichols, 2020). Volunteering can also create opportunities in a tight, competitive local job market (Babatunde-Sowole et al., 2020). Refugees with better resilience tend to be more involved in social initiatives in their local neighborhoods and volunteer with social organizations (Paloma et al., 2020).

###### Access to Opportunities

This factor is related to resources from the host society that refugees can access and benefit from; no evidence is available from quantitative research, but six qualitative studies have found it to be positively associated with resilience. Refugees have benefited from opportunities for employment and growth from formal organizations in the host country (Liu et al., 2020) and assistance from local non-profit organizations (Kuttikat et al., 2018). Knowledge and awareness of available resources has been shown to facilitate successful settlement and integration (Abur & Mphande, 2020; Corley & Sabri, 2020), including resources and social services, such as language classes.

###### Sense of Safety

The host society can offer a sense of safety, which is perceived as forming the basis for seeking out further opportunities for refugees. We identified this factor in six qualitative studies; it was not examined in quantitative research. In a U.S. study with Syrian refugees, safety was cited as beneficial for seeking employment and building a new life (Hasan et al., 2018). Feeling safe in the neighborhood and having access to natural spaces positively affected refugee women’s well-being (Liu et al., 2020). A sense of safety in the new country gave the freedom to choose the new opportunities granted by the host country (Aube et al., 2019) and yielded a feeling of comfort (Simich et al., 2010).

## Discussion

The aim of the present review is to synthesize existing knowledge on resilience in adult refugee populations. The research literature consists of studies that are highly diverse in terms of focus, location, design, outcomes, and measurements, including whether they measured resilience directly or via mental health problems as proxy indicators. Given this heterogeneity, we opted to use a narrative synthesis approach to review the studies. We organized the findings into socio-ecological levels to facilitate integration. Our review reveals clear risk factors: traumatic experiences and gender (being female) at the individual level and postmigration stress and unemployment at the societal level. We also identified protective factors, including social support at the family and community levels.

### Socio-Ecological Risk and Protective Factors Associated with Mental Health Problems and Resilience

Across individual, family, community, and societal levels, we identified 20 risk factors and 17 protective factors. Of the risk factors, 8 (40%) were located at the individual level, 3 (15%) at the family level, 2 (10%) at the community level, and 7 (35%) at the societal level. The distribution was quite similar for the protective factors: 8 (47%) were at the individual level, and 3 (17.6%) were at each of the family, community, and societal levels.

The variability in how resilience has been defined across studies presents a challenge to the integration of this body of research. To address this, we included studies that directly measured resilience using a resilience scale, as well as research that utilized measures of mental health problems as an inverse indicator of resilience. Most of the quantitative studies in our review used measures of mental health problems to index resilience outcomes. Only a few quantitative studies employed direct resilience scales. On the other hand, qualitative research tended to be exploratory, providing rich and in-depth narratives of a smaller number of individuals. Our synthesis of existing findings highlights several risk and protective factors for which findings are consistent despite these differences; it also identifies a number of potentially impactful factors that have yet to be thoroughly studied.

Table 3 displays factors contributing to refugees’ resilience at each socio-ecological level. It shows that individual and societal levels have been studied more, while family and community levels have received less attention. We next discuss the main findings at each level, including how the factors identified in our review map onto results from previous reviews in this research domain. Most of the studies in our review were conducted at the individual level, and unsurprisingly, our findings at this level are consistent with previous reviews. Important risk factors include traumatic experiences, being female, older, and having low SES (Bogic et al., 2015; Farahani et al., 2021; Scharpf et al., 2021), chronic physical and mental health issues (Scharpf et al., 2021), language difficulties (Bogic et al., 2015; Farahani et al., 2021; Hajak et al., 2021), and maladaptive coping (Scharpf et al., 2021). Similarly, most of our findings on protective factors at the individual level align with findings from previous reviews, including the role of positive coping (Tol et al., 2013), religiosity and spirituality (Babatunde-Sowole et al., 2016; Hawkes et al., 2020; Siriwardhana et al., 2014; Sleijpen et al., 2016), positive emotional experience (Sleijpen et al., 2016), and self-agency (Tol et al., 2013). However, our review additionally identifies higher education and language ability as protective factors and chronic physical health issues as risk factors; these factors have not been noted in previous reviews.

**Table 3.** Integration of Risk and Protective Factors of Resilience in Adult Refugees from Quantitative and Qualitative Studies Using the Socio-Ecological Framework.

Factors	Individual Level	Family Level	Community Level	Society Level
Risk factors	Traumatic experiences (98, 15*)	Living conditions (9)	Social isolation (4)	Postmigration stress (36, 14*)
	Being female (43)	Financial strain (5, 12*)	Community scrutiny* (7)	Unemployment (18, 13*)
	Being older (28) Chronic mental health issues (19)	Health problems or trauma of loved ones (4)		Perceived discrimination (14, 15*) Non-permanent legal status (12) Lack of professional recognition* (7)
	Chronic physical health issues (18, 5*)			Length of stay in refugee camps (7) Length of stay in the host country (6)
	Language difficulties (19, 7*) Low SES (10)			
	Maladaptive coping (8)			
Protective factors	Higher education (18) Positive coping (6, 23*) Religiosity (5, 30*)	Family support (9, 16*) Family-derived sense of purpose* (10)	Community support (8, 16*) Social relations (6, 17*)	Integration/acculturation (9, 13*) Access to opportunities* (6) Sense of safety* (6)
	Positive emotions* (17) Personality traits* (14)	Being married (12)	Ethnic/cultural identity (6, 17*)	
	Self-agency* (8) Spirituality* (5)			
	Self-efficacy* (4)			

*Note.* Each number in the brackets presents the number of studies with significant associations. Factors from quantitative studies are written without an asterisk; factors from qualitative studies are written with an asterisk. In bold are factors from both quantitative and qualitative studies. SES = socio-economic status.

On the family level, our review found that poor living conditions constitute a risk factor, consistent with a previous review (Farahani et al., 2021). Notably, we also identified financial strain and health problems or trauma experienced by loved ones as additional risk factors for resilience at the family level. We consider financial strain to be a risk factor for mental health problems at the family level because it occurs when people consider themselves unable to make ends meet for their families. Regarding the factor of health problems or trauma experienced by loved ones as a risk factor at the family level, studies suggest that having a family member suffering from a health problem or trauma can increase the likelihood of mental health problems in refugees. This connection implies that the health and trauma experiences of loved ones have a direct relationship with the resilience and mental health of individuals within the family unit. As a result, it is considered a family-level risk factor, given its significant implications on the mental health outcomes of family members. In terms of protective factors, we identified family support, being married, and a family-derived sense of purpose, with evidence from previous reviews of family support (Hawkes et al., 2020; Siriwardhana et al., 2014; Sleijpen et al., 2016; Tol et al., 2013) and being married (Bogic et al., 2015). A family-derived sense of purpose was not identified in previous reviews.

At the community and societal level, we found community scrutiny and social isolation to constitute risk factors and community support, social relations, and integration/acculturation to be protective factors. Our findings align with previous reviews, which found evidence supporting community scrutiny (Scharpf et al., 2021), community support (Babatunde-Sowole et al., 2016; Farahani et al., 2021; Marley & Mauki, 2018; Pieloch et al., 2016; Scharpf et al., 2021; Siriwardhana et al., 2014; Tol et al., 2013), social relations (Farahani et al., 2021), and integration/acculturation (Scharpf et al., 2021; Sleijpen et al., 2016) to be risk and protective factors of refugees’ resilience. In addition to these factors, our review also identified lack of professional status recognition, access to opportunities, and sense of safety as important factors affecting refugees’ resilience at the societal level. These factors were not identified in previous reviews, likely because they mainly focused on children and adolescents.

### Strength and Reliability of Identified Factors

The present review integrated findings from studies that measured resilience directly and indirectly, using a socio-ecological framework to group the factors at the individual, family, community, and society levels. We organized the factors derived from quantitative studies into four quadrants (see Supplemental Material 3), shown based on the frequency and consistency of the results pertaining to each factor. Since we tried to map the literature on the risk and protective factors of resilience, information about the frequency of the factors and the consistency of their results would be beneficial, that is, to understand the strength and reliability of the evidence base. This analysis focuses on quantitative studies only since most of the studies included in this review are quantitative, and it was possible to check the high or low consistency of the results. High-consistency factors exhibit a high level of agreement regarding their association and the direction with refugee resilience. Meanwhile, low, consistent factors exhibit a lower level of agreement or consensus among the available evidence.

The top left quadrant contains factors that have been extensively studied and consistently shown to be significant (high-frequency high-consistency factors). Factors in these clusters have attracted significant attention from researchers for a long time and consistently demonstrated their association with resilience across numerous studies. Besides, these factors are also perceived as significant predictors of resilience based on existing theoretical frameworks or models of resilience. Out of 171 quantitative studies, 100 (58.48%) were high quality, and 71 (41.52%) were low quality. Traumatic experiences are the most frequent and consistent factor, comprising 62 high-quality studies (62%) and 38 low-quality studies (38%). Being female consists of 30 high-quality (70%) and 13 low-quality (30%). Unemployment comprises 12 high-quality (63%) and 7 low-quality (37%) studies. Postmigration stress comprises 22 high-quality (61%) and 14 low-quality (39%) studies. Finally, social support comprises 12 high-quality (46%) and 14 low-quality (54%) studies.

The top right quadrant contains high-frequency low-consistency factors, that is, factors that have been widely studied but where studies have yielded inconsistent results. This applies to several demographic variables, including level of education, age, and marital status. While most studies have found no relationship between these factors and resilience, a significant relationship has been found in about one-third of the studies. Conflicting results for these factors may reflect variability in the postmigration situations in which the studies were conducted (Chung et al., 2020; Steel et al., 2006; Tinghög et al., 2017). For instance, refugees with higher education levels might suffer from greater status loss, which can, in some contexts, negate the positive effect that higher education can have in buffering the impact of displacement. Demographic variables in this quadrant may produce inconsistent results due to contextual influences, the possibility of moderating factors, and potential publication bias.

Regarding the lack of association between age and resilience, one study suggested that the narrow age range of their participants (30–45 years) might be the reason for the null finding (Carlsson et al., 2006). Another study had limitations regarding sample selection, with a very low mean age of respondents (Kaya et al., 2019). Moreover, the complexity of the relationship between demographic variables and resilience may also reflect the fact that some risk and protective factors may affect different demographic groups to varying degrees, with some populations being relatively spared from exposure to war-related stressors. The relationship between demographic variables and resilience can thus be expected to be complex (Miller, Weine, et al., 2002). In conclusion, although studies have extensively examined the impact of background variables like level of education, age, and marital status on resilience, results have been mixed, likely reflecting the complex ways in which they impact resilience. Future studies could additionally investigate how these factors interact with each other and other variables to predict resilience.

The bottom left quadrant displays low-frequency high-consistency factors. Despite being less studied, studies of these factors have yielded consistent results. Most of the factors examined in the present review fit into this group, for example, coping strategies, religiosity, cultural identity, integration/acculturation as the protective factors, language difficulties, physical and mental health problems, and SES as the risk factors. However, further research is needed, especially on factors that pertain to the family and community level, such as financial strain, health or trauma experienced by loved ones, a family-derived sense of purpose, and social isolation, given the limited evidence available on these factors.

The bottom right quadrant contains low-frequency low-consistency factors. We did not find any factors that would fit into this quadrant. It might be because of our inclusion criteria of the factors; as we mentioned in the “Results” section earlier, we only included factors for which associations with resilience were reported as statistically significant (*p* < .05) in more than three studies (Bogic et al., 2015). Studies with inconsistent findings or factors that have received limited attention may be excluded due to stringent inclusion criteria, thus reducing the likelihood of identifying low-frequency low-consistency factors.

Thus, our review has identified factors across various socio-ecological levels that constitute risk and protective factors for the resilience of refugees. In addition to strengthening the knowledge base on individual-level factors that have been widely studied, we also provide consolidating evidence for findings at the societal level. Our findings point to important sources of risk and protection for refugees’ resilience at the family and community level, including social support from family and community. However, additional research is needed to examine resilience at these levels to test the robustness of these findings across refugee populations.

### Limitations

This systematic scoping review has limitations that should be taken into consideration. A major limitation was the fact that a meta-analysis could not be conducted, precluding the possibility of calculating effect sizes for the different factors. This was due to the high methodological heterogeneity across studies, including differences in research designs, measurement tools, refugees’ characteristics, and resilience outcomes. For instance, studies used different tools to measure the same mental health outcomes; some used tools specific to the population, and others used established measurements that were either culturally validated or not. In terms of the settings of the studies and participants’ backgrounds, we acknowledge the limitation to generalizing the results of this review to the majority of refugees worldwide.

Statistics showed that 80% of refugees live in low- and middle-income countries, and only a minority live in high-income countries, (UNHCR, 2020). However, most studies have been conducted with refugees in high-income countries, and most of the factors identified in this review, especially on the community and society level (e.g., social support, acculturation, and postmigration stress), stem from these settings. In contrast, there is a striking lack of evidence on many of these factors from refugees living in low- and middle-income countries.

Another limitation is that most of the evidence in this review is based on cross-sectional research, making it difficult to establish causality between the factors and the outcomes. Furthermore, self-report instruments used in the cross-sectional research capture subjective perceptions of various factors, including support, opportunities, discrimination, and postmigration stress. This method has the inherent limitation that the respondent’s current mental health state or well-being may bias their perceptions and responses. Such subjectivity could influence the interpretation of the directionality and strength of the relationships between these factors. Finally, while we drew on evidence from both quantitative and qualitative studies for most of the relationships we examined, this was not the case for every factor. This hampers the robustness of the conclusions. Despite these limitations, we believe that these findings can nevertheless provide valuable insights that can guide the design of future studies or interventions to improve resilience among refugees.

### Implications for Research on Refugees’ Resilience

The evidence we have reviewed suggests that family and community are essential for the resilience of refugees, but studies have yet to investigate these factors in depth (see Table 4 for a summary of the implications). Further research is needed, particularly on the factors that have yielded highly consistent results at these levels. These include living conditions, financial strain, health and trauma experienced by loved ones, social isolation, social relations, integration/acculturation, and cultural identity. However, it is also worth examining high-frequency high-consistency factors, such as social support, but in a different approach, that is, to differentiate it with the type and source of support across socio-ecological levels.

**Table 4.** Summary of Implications for Policy.

Summary of Key Implications
Family and community support are crucial for refugee resilience, but there is a lack of in-depth research on these factors.
Further research is needed, especially on consistent factors like living conditions, financial strain, health, trauma, social isolation, social relations, integration, and cultural identity.
High-frequency, high-consistency factors like social support should be examined with a focus on the type and source of support across different socio-ecological levels.
Longitudinal studies with moderator and mediator variables on different levels are necessary.
Some factors are less changeable (e.g., gender, pre-resettlement traumatic experiences), while others are more flexible (e.g., living conditions and financial strain).
Trauma-focused interventions are crucial for adult refugees, especially women, due to the lasting impact of traumatic experiences.
Family support is essential for refugees’ resilience, emphasizing the need for family reunification programs to provide crucial family-level support.
At the community level, both risk factors (e.g., community scrutiny) and protective factors (e.g., social support) are important, highlighting the need for supportive communities throughout the asylum and integration process.
Policymakers should address risk factors like postmigration stress and unemployment at the societal level.
Tailored integration programs and recognition of refugees’ professional qualifications can help them integrate into local labor markets successfully.

More research is needed to examine the interaction between factors on different levels, particularly with moderator and mediator variables. For example, a recent study by Pak et al. (2022) found that self-efficacy mediated the relationship between social support and resilience among Syrian refugees living in Turkey. Getnet et al. (2019) tested mediating and moderating factors in the relationship between trauma exposure and psychological symptoms among Eritrean refugees in Ethiopia. They found that a sense of coherence and a task-oriented coping style partially mediated the association between trauma and PTSD symptoms. Emotion-oriented coping style and social support moderated the effect of premigration and postmigration living difficulties. In a study conducted by Stempel et al. (2016), the impact of various sources of distress and resilience on Afghan refugees in Northern California was explored, focusing on how gender influenced and mediated these factors. The study revealed significant gender differences in the type and impact of resettlement stressors and sources of resilience.

In addition, our review points to factors that may play a role in intervention studies. Of course, some factors are less malleable (e.g., gender and traumatic experiences occurring before resettlement), while others are more so (e.g., living conditions and financial strain). Specifically, it makes sense to target interventions aimed at high-frequency high-consistency risk factors, such as postmigration stress. Providing support and resources (e.g., bilingual mental health professionals and vocational training programs) to help refugees manage postmigration stress and build resilience holds great promise for future interventions. Finally, we recognize the importance of incorporating promotive factors—those contributing to well-being and positive outcomes—into conceptualizing resilience as a process. While we agree that including these factors would provide a more holistic view, we cannot incorporate this aspect into the current manuscript due to time and space constraints. We recommend that future studies address this important facet of resilience.

### Implications for Policy

The practical implications of this review aim to guide policy and intervention targeting vulnerable refugee populations by considering factors that contribute to their resilience across socio-ecological levels. Trauma-focused interventions are essential for adult refugees, particularly women, given the pervasive impact of traumatic experiences. However, it is crucial to consider cultural identity when designing these interventions, as we found cultural aspects to be important for refugees’ resilience. Our results point to family support being crucial to refugees’ resilience, highlighting the importance of family reunification programs. At the community level, both risk factors (e.g., community scrutiny) and protective factors (e.g., social support) were identified, pointing to the importance of scaffolding supportive communities for refugees throughout the asylum and integration process. We recommend the development of community-based intervention programs, with particular emphasis on opportunities for social support. At the societal level, postmigration stress and unemployment are established risk factors that policymakers can aim to address. Integration programs tailored to individual needs can additionally help refugees gain relevant qualifications, as can enhanced systems for recognizing refugees’ existing professional qualifications to facilitate their integration into local labor markets.

## Conclusions

Multiple factors across socio-ecological domains are associated with refugees’ resilience. The current review demonstrates a solid evidence base for several individual-level factors, such as traumatic experiences and gender (being female), and societal-level factors, such as postmigration stress and unemployment. Moreover, protective factors were identified at the family and community levels in the form of social support. It is our hope that these findings will spur further research and provide insights into the use of policies and interventions to strengthen the resilience of refugees.

## Supplemental Material

sj-docx-1-tva-10.1177_15248380241284594 – Supplemental material for The Socio-Ecological Factors Associated with Mental Health Problems and Resilience in Refugees: A Systematic Scoping Review Supplemental material, sj-docx-1-tva-10.1177_15248380241284594 for The Socio-Ecological Factors Associated with Mental Health Problems and Resilience in Refugees: A Systematic Scoping Review by Tengku Nila Fadhlia, Bertjan Doosje and Disa A. Sauter in Trauma, Violence, & Abuse

sj-docx-2-tva-10.1177_15248380241284594 – Supplemental material for The Socio-Ecological Factors Associated with Mental Health Problems and Resilience in Refugees: A Systematic Scoping Review Supplemental material, sj-docx-2-tva-10.1177_15248380241284594 for The Socio-Ecological Factors Associated with Mental Health Problems and Resilience in Refugees: A Systematic Scoping Review by Tengku Nila Fadhlia, Bertjan Doosje and Disa A. Sauter in Trauma, Violence, & Abuse

sj-docx-3-tva-10.1177_15248380241284594 – Supplemental material for The Socio-Ecological Factors Associated with Mental Health Problems and Resilience in Refugees: A Systematic Scoping Review Supplemental material, sj-docx-3-tva-10.1177_15248380241284594 for The Socio-Ecological Factors Associated with Mental Health Problems and Resilience in Refugees: A Systematic Scoping Review by Tengku Nila Fadhlia, Bertjan Doosje and Disa A. Sauter in Trauma, Violence, & Abuse

sj-docx-4-tva-10.1177_15248380241284594 – Supplemental material for The Socio-Ecological Factors Associated with Mental Health Problems and Resilience in Refugees: A Systematic Scoping Review Supplemental material, sj-docx-4-tva-10.1177_15248380241284594 for The Socio-Ecological Factors Associated with Mental Health Problems and Resilience in Refugees: A Systematic Scoping Review by Tengku Nila Fadhlia, Bertjan Doosje and Disa A. Sauter in Trauma, Violence, & Abuse

sj-docx-5-tva-10.1177_15248380241284594 – Supplemental material for The Socio-Ecological Factors Associated with Mental Health Problems and Resilience in Refugees: A Systematic Scoping Review Supplemental material, sj-docx-5-tva-10.1177_15248380241284594 for The Socio-Ecological Factors Associated with Mental Health Problems and Resilience in Refugees: A Systematic Scoping Review by Tengku Nila Fadhlia, Bertjan Doosje and Disa A. Sauter in Trauma, Violence, & Abuse

## References

[bibr1-15248380241284594] We put a reference list of the studies included in our review in the Supplemental Material for this project on OSF: https://osf.io/b79ph/?view_only=86f5db51bdb74e28ba1a29e1aaaa1e71

[bibr2-15248380241284594] Babatunde-Sowole O. Power T. Jackson D. Davidson P. M. Digiacomo M. (2016). Resilience of African migrants: An integrative review. Health Care for Women International, 37(9), 946–963. 10.1080/07399332.2016.1158263 26930120

[bibr3-15248380241284594] Bogic M. Njoku A. Priebe S. (2015). Long-term mental health of war-refugees: A systematic literature review. BMC International Health and Human Rights, 15, 1–41. 10.1186/s12914-015-0064-9 26510473 PMC4624599

[bibr4-15248380241284594] Bronfenbrenner U. (1979). The ecology of human development: Experiment by nature and design. MA London Harvard University Press.

[bibr5-15248380241284594] Cooper C. Katona C. Orrell M. Livingston G. (2006). Coping strategies and anxiety in caregivers of people with Alzheimer’s disease: The LASER-AD study. J Affect Disord, 90, 15–20. 10.1016/j.jad.2005.08.017 16337688

[bibr6-15248380241284594] Dangmann C. Solberg Ø. Myhrene Steffenak A. K. Høye S. Andersen P. N. (2021). Syrian fefugee youth resettled in Norway: Mechanisms of resilience influencing health-related quality of life and mental distress. Frontiers in Public Health, 9, 1–13. 10.3389/fpubh.2021.711451 PMC8494783 34631646

[bibr7-15248380241284594] Farahani H. Joubert N. Anand J. C. Toikko T. Tavakol M. (2021). A systematic review of the protective and risk factors influencing the mental health of forced migrants: Implications for sustainable intercultural mental health practice. Social Sciences, 10, 1–24.

[bibr8-15248380241284594] Fletcher D. Sarkar M. (2013). Psychological resilience: A review and critique of definitions, concepts, and theory. European Psychologist, 18(1), 12–23.

[bibr9-15248380241284594] Frounfelker R. L. Mishra T. Carroll A. Brennan R. T. Gautam B. Ali E. A. A. Betancourt T. S. (2022). Past trauma, resettlement stress, and mental health of older Bhutanese with a refugee life experience. Aging and Mental Health, 26(11), 2149–2158. 10.1080/13607863.2021.1963947 34396853 PMC9386683

[bibr10-15248380241284594] Getnet B. Medhin G. Alem A. (2019). Symptoms of post-traumatic disorder and depression among Eritrean refugees in Ethiopia: Identifying direct, mediating and moderating predictors from path analysis. BMJ Open, 2, 1–12. 10.1136/bmjopen-2017-021142 PMC6340455 30659034

[bibr11-15248380241284594] Haddaway N. R. Page M. J. Pritchard C. C. McGuinness L. A. (2022). PRISMA2020: An R package and Shiny app for producing PRISMA 2020-compliant flow diagrams, with interactivity for optimised digital transparency and Open Synthesis. Campbell Systematic Reviews, 18(2), 1–12. 10.1002/cl2.1230 PMC8958186 36911350

[bibr12-15248380241284594] Hajak V. L. Sardana S. Verdeli H. Grimm S. (2021). A systematic review of factors affecting mental health and well-being of asylum seekers and refugees in Germany. Frontiers in Psychiatry, 12(12), 1–15. 10.3389/fpsyt.2021.643704 PMC8012840 33815176

[bibr13-15248380241284594] Hassan G. Ventevogel P. Jefee-Bahloul H. Barkil-Oteo A. Kirmayer L. J. (2016). Mental health and psychosocial wellbeing of Syrians affected by armed conflict. Epidemiology and Psychiatric Sciences, 25, 129–141. 10.1017/S2045796016000044 26829998 PMC6998596

[bibr14-15248380241284594] Hawkes C. Norris K. Joyce J. Paton D. (2020). Resilience factors in women of refugee background: A qualitative systematic review. Community Psychology in Global Perspective, 6(2–1), 101–127. 10.1285/i24212113v6i2-1p101

[bibr15-15248380241284594] Henderson D. X. DeCuir-Gunby J. Gill V. (2016). “It really takes a village”: A socio-ecological model of resilience for prevention among economically disadvantaged ethnic minority youth. Journal of Primary Prevention, 37(5), 469–485. 10.1007/s10935-016-0446-3 27624607

[bibr16-15248380241284594] Ipekci B. (2018). Complexity of resiliency framework for refugee population: A letter to the editor regarding Wright et al. (2016). Journal of Immigrant and Minority Health, 20(5), 1041. 10.1007/s10903-018-0698-5 29350319

[bibr17-15248380241284594] Lewis D. Rödlach A. (2019). Introduction to the special issue: Resilience and wellbeing in forced migration. Migration Letters, 16(3), 355–359. 10.33182/ml.v16i3.637

[bibr18-15248380241284594] Lindert J. (2017). Psychological resilience in refugees—A systematic review. European Journal of Public Health, 27, 185. 10.1093/eurpub/ckx187.473

[bibr19-15248380241284594] Marley C. Mauki B. (2018). Resilience and protective factors among refugee children post-migration to high-income countries: A systematic review. European Journal of Public Health, 29(4), 706–713. 10.1093/eurpub/cky232 30380016

[bibr20-15248380241284594] Masten A. S. (2001). Ordinary magic: Resilience processes in development. American Psychologist, 56(3), 227–238. 11315249 10.1037//0003-066x.56.3.227

[bibr21-15248380241284594] Ouzzani M. Hammady H. Fedorowicz Z. Elmagarmid A. (2016). Rayyan—A web and mobile app for systematic reviews. Systematic Reviews, 5(1), 210. 10.1186/S13643-016-0384-4 27919275 PMC5139140

[bibr22-15248380241284594] Petticrew M. Roberts H. (2006). Systematic Reviews in the Social Sciences: A Practical Guide. Australia: Blackwell Publishing.

[bibr23-15248380241284594] Pieloch K. A. McCullough M. B. Marks A. K. (2016). Resilience of children with refugee statuses: A research review. Canadian Psychology, 57(4), 330–339. 10.1037/cap0000073

[bibr24-15248380241284594] Polat S. Kröner S. (2022). The resilience of school-age immigrant children: A scoping review. Journal of Human Behavior in the Social Environment, 33(3), 329–347. 10.1080/10911359.2022.2061664

[bibr25-15248380241284594] Scharpf F. Kaltenbach E. Nickerson A. Hecker T. (2021). A systematic review of socio-ecological factors contributing to risk and protection of the mental health of refugee children and adolescents. Clinical Psychology Review, 83, 101930. 10.1016/j.cpr.2020.101930 33186775

[bibr26-15248380241284594] Siriwardhana C. Ali S. S. Roberts B. Stewart R. (2014). A systematic review of resilience and mental health outcomes of conflict-driven adult forced migrants. Conflict and Health, 8(1), 13. 10.1186/1752-1505-8-13 25177360 PMC4149800

[bibr27-15248380241284594] Siriwardhana C. Stewart R. (2013). Forced migration and mental health: Prolonged internal displacement, return migration and resilience. International Health, 5(1), 19–23. 10.1093/inthealth/ihs014 24029841

[bibr28-15248380241284594] Sleijpen M. Boeije H. R. Kleber R. J. Mooren T. (2016). Between power and powerlessness: A meta-ethnography of sources of resilience in young refugees. Ethnicity and Health, 21(2), 158–180. 10.1080/13557858.2015.1044946 26107385

[bibr29-15248380241284594] Steel Z. Momartin S. Silove D. Coello M. Aroche J. Tay K. W. (2011). Two year psychosocial and mental health outcomes for refugees subjected to restrictive and supportive migration policies. Social Science & Medicine, 72, 1149–1156. http://dx.doi.org/10.1016/j.socscimed.2011.02.007 21427011 10.1016/j.socscimed.2011.02.007

[bibr30-15248380241284594] Tol W. A. Song S. Jordans M. J. D. (2013). Annual Research Review: Resilience and mental health in children and adolescents living in areas of armed conflict—A systematic review of findings in low- and middle-income countries. Journal of Child Psychology and Psychiatry, 54(4), 445–460. 10.1111/jcpp.12053 23414226

[bibr31-15248380241284594] Tricco A. C. Lillie E. Zarin W. O’Brien K. K. Colquhoun H. Levac D. Moher D. Peters M. D. J. Horsley T. Weeks L. Hempel S. Akl E. A. Chang C. McGowan J. Stewart L. Hartling L. Aldcroft A. Wilson M. G. Garritty C. , . . . Straus S. E. (2018). PRISMA Extension for Scoping Reviews (PRISMA-ScR): Checklist and explanation. Annals of Internal Medicine, 169(7), 467–473. 10.7326/M18-0850 30178033

[bibr32-15248380241284594] Ungar M. (2011). The social ecology of resilience: Addressing contextual and cultural ambiguity of a nascent construct. American Journal of Orthopsychiatry, 81(1), 1–17. 10.1111/j.1939-0025.2010.01067.x 21219271

[bibr33-15248380241284594] Ungar M. (2018). Systemic resilience: Principles and processes for a science of change in contexts of adversity. Ecology and Society, 23(4), 34–50. 10.5751/ES-10385-230434

[bibr34-15248380241284594] Ungar M. Theron L. (2019). Resilience and mental health: How multisystemic processes contribute to positive outcomes. The Lancet Psychiatry, 7(5), 441–448. 10.1016/S2215-0366(19)30434-1 31806473

[bibr35-15248380241284594] United Nations High Commissioner for Refugees (UNHCR). (2006). Master glossary of terms Rev. 1 (Vol. 1). Division of International Protection Services. https://www.refworld.org/cgi-bin/texis/vtx/rwmain?docid=42ce7d444

[bibr36-15248380241284594] United Nations High Commissioner for Refugees (UNHCR). (2020). Forced displacement passes 80 million by mid-2020 as COVID-19 tests refugee protection globally. https://www.unhcr.org/news/news-releases/forced-displacement-passes-80-million-mid-2020-covid-19-tests-refugee-protection

[bibr37-15248380241284594] United Nations High Commissioner for Refugees(UNHCR). (2022). Refugee data finder. https://www.unhcr.org/refugee-statistics/

[bibr38-15248380241284594] Verbeek J. Ruotsalainen J. Hoving J. L. (2012). Synthesizing study results in a systematic review. Scandinavian Journal of Work, Environment and Health, 38(3), 282–290. 10.5271/sjweh.3201 22015561

[bibr39-15248380241284594] VERBI GmbH. (2020). MAXQDA | Software for qualitative and mixed methods research. https://www.maxqda.com/what-is-maxqda

