# Supplementary material for: The Socio-Ecological Factors Associated with Mental Health Problems and Resilience in Refugees: A Systematic Scoping Review
Source: Trauma Violence Abuse. 2024 Oct 8;26(3):598–616. doi: 10.1177/15248380241284594 (PMC12145474; doi:10.1177/15248380241284594)
Supplement: sj-docx-1-tva-10.1177_15248380241284594 – Supplemental material for The Socio-Ecological Factors Associated with Mental Health Problems and Resilience in Refugees: A Systematic Scoping Review [file sj-docx-1-tva-10.1177_15248380241284594.docx]

**Supplemental Material 1**

**Search Strategy (First Round)**

The searches on **February 12, 2021** below yielded the following results:

**Databases**

PsycINFO 3784 results

Web of Science 5817 results

SocINDEX 1523 results

Total 11.124 results (8271, 7899 after deduplication)

**PsycINFO**

*Ovid, APA PsycInfo 1806 to February 12, 2021*

**#1 Resilience**

"resilience (psychological)"/ OR adversity/ OR protective factors/ OR posttraumatic growth/ OR psychological endurance/ OR coping behavior/ OR mental health/ OR mental wellbeing/ OR psychological stress/ OR emotional adjustment OR (resilien* OR protective factor* OR risk factor* OR mental health outcome* OR posttrauma* growth OR post-traumatic growth OR psychological endurance OR coping OR depression OR posttraumatic stress disorder OR anxiety OR stress OR distress OR adversity).ti,ab,id.

**#2 refugees**

refugees/ OR asylum seeking/ OR (refugee* OR asylum* seek* OR displaced people OR forced migrant* OR irregular migrant*).ti,ab,id.

**1 AND 2 3784 results**

**Web of Science Core Collection**

*Web of Science*

**#1 Resilience**

TS=("resilien*" OR "protective factor*" OR "risk factor*" OR "mental health" OR "posttrauma* growth" OR "post-traumatic growth" OR "psychological endurance" OR "coping" OR "adversity" OR "mental wellbeing" OR "psychological stress" OR "emotional adjustment" OR "mental health outcome*" OR "depression" OR "posttraumatic stress disorder" OR "anxiety" OR "stress" OR "distress")

**#2 refugees**

TS=("refugee*" OR "asylum* seek*" OR "displaced people" OR "forced migrant*" OR "irregular migrant*")

**1 AND 2 5.817 results**

NB. It is not possible here to safely exclude children from the search results

**SocINDEX**

*EBSCO*

**#1 Resilience**

TI("resilien*" OR "protective factor*" OR "risk factor*" OR "mental health" OR "posttrauma* growth" OR "post-traumatic growth" OR "psychological endurance" OR "coping" OR "adversity" OR "mental wellbeing" OR "psychological stress" OR "emotional adjustment" OR "mental health outcome*" OR "depression" OR "posttraumatic stress disorder" OR "anxiety" OR "stress" OR "distress") OR AB("resilien*" OR "protective factor*" OR "risk factor*" OR "mental health" OR "posttrauma* growth" OR "post-traumatic growth" OR "psychological endurance" OR "coping" OR "adversity" OR "mental wellbeing" OR "psychological stress" OR "emotional adjustment" OR "mental health outcome*" OR "depression" OR "posttraumatic stress disorder" OR "anxiety" OR "stress" OR "distress" ) OR KW("resilien*" OR "protective factor*" OR "risk factor*" OR "mental health" OR "posttrauma* growth" OR "post-traumatic growth" OR "psychological endurance" OR "coping" OR "adversity" OR "mental wellbeing" OR "psychological stress" OR "emotional adjustment" OR "mental health outcome*" OR "depression" OR "posttraumatic stress disorder" OR "anxiety" OR "stress" OR "distress")

**#2 refugees**

TI("refugee*" OR "asylum* seek*" OR "displaced people" OR "forced migrant*" OR "irregular migrant*") OR AB("refugee*" OR "asylum* seek*" OR "displaced people" OR "forced migrant*" OR "irregular migrant*") OR KW("refugee*" OR "asylum* seek*" OR "displaced people" OR "forced migrant*" OR "irregular migrant*")

**#3 adults (not children)**

(DE ("child*" OR "adolesc*" OR "teenag*" OR "youth")) NOT (DE "adults" OR "middle aged*")

**S1 AND S2 1.754 results**

**(S1 AND S2) NOT S3 1.523 results**

**Reference Check**

Resilience and mental health risks among Syrian refugees in Europe: A cultural perspective

Social Determinants of Resilience Among Syrian Refugees in Jordan

And Then the War Came: A Content Analysis of Resilience Processes in the Narratives of Refugees from Humans of New York

Resilience among Single adult female refugees in Hamilton, Ontario

Searching for Tomorrow—South Sudanese Women Reconstructing Resilience through Photovoice

Coping and resilience in refugees from the Sudan: A narrative account

The searches on **June 10, 2022** yielded the following results:

**Databases**

PsycINFO 498 results

Web of Science 1.455 results

SocINDEX 157 results

Total 2.110 results (1.768 results after deduplication)

**PsycINFO**

*Ovid, APA PsycInfo 2021 to June 10, 2022*

**#1 Resilience**

"resilience (psychological)"/ OR adversity/ OR protective factors/ OR posttraumatic growth/ OR psychological endurance/ OR coping behavior/ OR mental health/ OR mental wellbeing/ OR psychological stress/ OR emotional adjustment OR (resilien* OR protective factor* OR risk factor* OR mental health outcome* OR posttrauma* growth OR post-traumatic growth OR psychological endurance OR coping OR depression OR posttraumatic stress disorder OR anxiety OR stress OR distress OR adversity).ti,ab,id.

**#2 refugees**

refugees/ OR asylum seeking/ OR (refugee* OR asylum* seek* OR displaced people OR forced migrant* OR irregular migrant*).ti,ab,id.

**1 AND 2 498 results**

**Web of Science Core Collection**

*Web of Science (Publication date: 13-02-2021 to 10-06-2022)*

**#1 Resilience**

TS=("resilien*" OR "protective factor*" OR "risk factor*" OR "mental health" OR "posttrauma* growth" OR "post-traumatic growth" OR "psychological endurance" OR "coping" OR "adversity" OR "mental wellbeing" OR "psychological stress" OR "emotional adjustment" OR "mental health outcome*" OR "depression" OR "posttraumatic stress disorder" OR "anxiety" OR "stress" OR "distress")

**#2 refugees**

TS=("refugee*" OR "asylum* seek*" OR "displaced people" OR "forced migrant*" OR "irregular migrant*")

**1 AND 2 1.455 results**

NB. It is not possible here to safely exclude children from the search results

**SocINDEX**

*EBSCO (publication year: 2021-2022)*

**#1 Resilience**

TI("resilien*" OR "protective factor*" OR "risk factor*" OR "mental health" OR "posttrauma* growth" OR "post-traumatic growth" OR "psychological endurance" OR "coping" OR "adversity" OR "mental wellbeing" OR "psychological stress" OR "emotional adjustment" OR "mental health outcome*" OR "depression" OR "posttraumatic stress disorder" OR "anxiety" OR "stress" OR "distress") OR AB("resilien*" OR "protective factor*" OR "risk factor*" OR "mental health" OR "posttrauma* growth" OR "post-traumatic growth" OR "psychological endurance" OR "coping" OR "adversity" OR "mental wellbeing" OR "psychological stress" OR "emotional adjustment" OR "mental health outcome*" OR "depression" OR "posttraumatic stress disorder" OR "anxiety" OR "stress" OR "distress" ) OR KW("resilien*" OR "protective factor*" OR "risk factor*" OR "mental health" OR "posttrauma* growth" OR "post-traumatic growth" OR "psychological endurance" OR "coping" OR "adversity" OR "mental wellbeing" OR "psychological stress" OR "emotional adjustment" OR "mental health outcome*" OR "depression" OR "posttraumatic stress disorder" OR "anxiety" OR "stress" OR "distress")

**#2 refugees**

TI("refugee*" OR "asylum* seek*" OR "displaced people" OR "forced migrant*" OR "irregular migrant*") OR AB("refugee*" OR "asylum* seek*" OR "displaced people" OR "forced migrant*" OR "irregular migrant*") OR KW("refugee*" OR "asylum* seek*" OR "displaced people" OR "forced migrant*" OR "irregular migrant*")

**#3 adults (not children)**

(DE ("child*" OR "adolesc*" OR "teenag*" OR "youth")) NOT (DE "adults" OR "middle aged*")

**S1 AND S2 183 results**

**(S1 AND S2) NOT S3 157 results**

**Reference Check**

Resilience and mental health risks among Syrian refugees in Europe: A cultural perspective

Social Determinants of Resilience Among Syrian Refugees in Jordan

And Then the War Came: A Content Analysis of Resilience Processes in the Narratives of Refugees from Humans of New York

Resilience among Single adult female refugees in Hamilton, Ontario

Searching for Tomorrow—South Sudanese Women Reconstructing Resilience through Photovoice

Coping and resilience in refugees from the Sudan: A narrative account
