# Supplementary material for: The Socio-Ecological Factors Associated with Mental Health Problems and Resilience in Refugees: A Systematic Scoping Review
Source: Trauma Violence Abuse. 2024 Oct 8;26(3):598–616. doi: 10.1177/15248380241284594 (PMC12145474; doi:10.1177/15248380241284594)
Supplement: sj-docx-2-tva-10.1177_15248380241284594 – Supplemental material for The Socio-Ecological Factors Associated with Mental Health Problems and Resilience in Refugees: A Systematic Scoping Review [file sj-docx-2-tva-10.1177_15248380241284594.docx]

**Supplemental Material 2: Coding Scheme**

| Categories | Parents Code | Sub codes | Sub codes 2 | Remarks/ Definition |
| --- | --- | --- | --- | --- |
| Purpose of the study |  |  |  |  |
| Type of study | Quantitative | Cross-sectional |  | Studies that collect data at a single point time from a sample of individuals or groups from different levels of the variables being studies. |
|  |  | Longitudinal |  | A design that follows group of individuals over a period of time to examine changes in the variables. |
|  |  | Experiment |  | A design in which researchers manipulate one or more independent variables to observe the effect on dependent variable, while controlling for other factors that could potentially influence the outcomes. |
|  | Qualitative |  |  | Any type of approach in qualitative study |
| Year of publication |  |  |  |  |
| Main finding |  |  |  | What is the main finding of the paper? Usually it’s stated on the abstract. |
| Data collection method | Resilience outcomes measurement |  |  | For quantitative studies: scale, questionnaire, and any kind of direct measurement they use to determine resilience outcomes.  For qualitative studies it could be interview, FGD, observational field notes, etc. |
|  | Mental health outcomes measurement |  |  | For quantitative studies: scale, questionnaire, and any kind of measurement they use to determine mental health outcomes.  For qualitative studies it could be interview, FGD, observational field notes, etc. |
| Statistical test |  |  |  | What kind of statistical analysis the author used to test the relationship between predictors and outcomes? |
| Samples | Middle-Eastern refugees |  |  | For examples: Syrians, Palestinians, Iraqis, Iranian, etc. |
|  | African refugees |  |  | For examples: Eritrean, Sudanese, Congolese, etc. |
|  | Central American refugees |  |  | For examples: Guatemalans, Salvadorians, etc. |
|  | East Asian refugees |  |  | For examples: North Koreans |
|  | South Asian refugees |  |  | For examples: Burmese, Sri Lankans |
|  | The Balkans refugees |  |  | For examples: Bosnians |
|  | The Caribbean refugees |  |  | For examples: Haitians |
|  | Others (please specify) |  |  | Samples that do not come from specific countries that have been listed above. |
|  | Refugees from various countries |  |  | Samples that come from multiple countries |
| Sample size | <10 |  |  |  |
|  | 11-50 |  |  |  |
|  | 51-100 |  |  |  |
|  | 101-200 |  |  |  |
|  | 201-500 |  |  |  |
|  | >500 |  |  |  |
| Age range of the samples |  |  |  |  |
| Sample selection | Random |  |  | Samples are selected randomly. |
|  | Snowball |  |  | Samples are selected like a snowball effect, e.g., participant 1 refer other participants. |
|  | Purposive |  |  | Samples are selected based on specific criteria that are relevant to research question. |
|  | Convenience |  |  | Samples are selected based on their availability and accessibility. |
|  | Mixed: Purposive and snowball |  |  |  |
|  | Mixed: Convenience and purposive |  |  |  |
|  | Mixed: Convenience and snowball |  |  |  |
|  | Others (please specify) |  |  |  |
| Region of the study | Europe (including the UK) |  |  | Where the study took place |
|  | North America/ Canada |  |  |  |
|  | Latin America and the Caribbean |  |  |  |
|  | Africa |  |  |  |
|  | Australia (including New Zealand) |  |  |  |
|  | Middle East |  |  |  |
|  | South Asia |  |  |  |
|  | East Asia |  |  |  |
|  | Others (please specify) |  |  |  |
| Outcomes | Resilience |  |  | Resilience outcomes as measured by the new or already existed resilience scale or based on the interview results for qualitative study. |
|  | Mental health problems | PTSD |  | We limit to four outcomes:  Depression, stress/distress, anxiety, and PTSD. It doesn’t have to be all of it, could be just one or two (whatever appear on the papers). These outcomes must be stated clearly in the result section and based on the measurement tools that have been stated in method section. |
|  |  | Depression |  |  |
|  |  | Anxiety |  |  |
|  |  | Psychological distress |  |  |
| Protective factors | Religiosity |  |  | Religious belief or faith to God or certain religion. |
|  | Spirituality |  |  | Belief in something bigger other than God and not associated with any certain religion. |
|  | Social support |  |  | Including family and community support. |
|  | Social relation |  |  | The interaction that occur with social network member (i.e., community member) |
|  | Digital support |  |  | For example: Facebook group, social media friend or interaction |
|  | Self-efficacy |  |  | a personal judgment of "how well one can execute courses of action required to deal with prospective situations". |
|  | Self-agency |  |  | Refers to the feeling of control over actions and their consequences. |
|  | Self-knowledge | Body knowledge and movement |  | Understanding of one’s own capabilities, character, feelings, or motivations |
|  | Economic empowerment |  |  | Being economically empowered. |
|  | Positive emotion |  |  | Having hope and positive outlook for the future, gratitude, joy, etc. |
|  | Sense of coherence |  |  | A mixture of optimism combined with a sense of control. When we have sense of coherence, we see the world as something that is manageable, understandable, and meaningful. |
|  | Coping-strategies | Emotion-focused |  | Coping strategies that are directed to deal with emotional effect from the problem, such as mindfulness experience, comparison with others, cognitive reframing, exercise and being active, structured daily activities, and artistic expression. |
|  |  | Problem-focused | Set goals | Coping strategies that are directed to solve the source problem, including setting goals. |
|  | Contributing to new community |  |  | Something that refugees do for the host country, such as voluntary work, activism, etc. |
|  | Access to opportunities |  |  | Resources from a society that refugees can access and benefit from, such as information access, social services, language class, training program, and access to health care system. |
|  | Sense of safety |  |  | Society offered a sense of safety and was perceived as forming the basis of seeking out opportunities provided by the new country. For example, feeling safe in the neighborhood. |
|  | Cultural identity |  |  | Including maintaining cultural identity, strong ethnic identity identification, and confidence in ethnic identity. |
|  | Integration/ acculturation |  |  | Process of being a part of the host country, such as participating in integration course, sociocultural adaptation, learning the language, and orientation toward host culture. |
|  | Personality traits |  |  | For example: persistence, perseverance, innovative, initiative, strong, determination, other personality characteristics of individual. |
|  | Demographic | Age |  | Older age and younger age. |
|  |  | Gender |  | Measured binary: men and women. |
|  |  | Socioeconomic status (SES) |  | Mostly measured by income: high SES and low SES. |
|  | Others (please specify) |  |  |  |
| Risk factors | Traumatic experiences |  |  | Premigration and migration traumas, such as separation from families, war experience, torture, persecution, etc. |
|  | Perceived discrimination |  |  | Discrimination as refugees perceived based on the discrimination scale. |
|  | Postmigration stress |  |  | Stress that emerges in postmigration period, such as acculturation stress, culture shock, climate shock, etc. |
|  | Language difficulties |  |  | The difficulties in learning and mastering language of the host country. |
|  | Having a downward job |  |  | The job that they have now is in different position or inferior to the one held before leaving the country of origin |
|  | Financial strain |  |  | Inability to make ends meet, pay the monthly bills, and provide for the family. |
|  | Lack of recognition of their pre-existing qualifications. |  |  |  |
|  | Unsafe environment |  |  |  |
|  | Safety issues |  |  | Including police extortion, harassment, arrests, deportations, and working illegally. |
|  | Community scrutiny |  |  | Besides being a protective factor, the refugees community could give some stressors and negative influences on refugees. For example, unhealthy competition, gossip within the community, jealousy, scrutiny, suspicion, and stigmatization. |
|  | Demographic | Age |  | Older age and younger age. |
|  |  | Gender |  | Measured binary: men and women. |
|  |  | SES |  | Mostly measured by income: high SES and low SES. |
|  | Chronic physical health issues |  |  | Long term physical health problems. |
|  | Chronic mental health issues |  |  | Long term mental health problems. |
|  | Maladaptive coping |  |  | The use of coping strategies or behaviors that do not effectively address a stressful situation, and may even create additional problems (e.g., denial, self-blame, self-distraction, substance use) |
|  | Others (please specify) |  |  |  |
