# Supplementary material for: The Socio-Ecological Factors Associated with Mental Health Problems and Resilience in Refugees: A Systematic Scoping Review
Source: Trauma Violence Abuse. 2024 Oct 8;26(3):598–616. doi: 10.1177/15248380241284594 (PMC12145474; doi:10.1177/15248380241284594)

**Supplemental Material 3**

*Consistency of Factors from Quantitative Studies. Factors are grouped by the socio-ecological level. Raw number followed by percentage in bracket are displayed per column for total number of studies reported significant results, total number of studies that tested the factors the directions of the results.*

| Level | Factor | Number of studies that reported significant results for the factor | Number of studies that tested the factor | Results | | | | |
| --- | --- | --- | --- | --- | --- | --- | --- | --- |
|  |  |  |  | Positive correlation with mental health problems | Positive correlation with resilience | Negative correlation with mental health problems | Negative correlation with resilience | No correlation |
| Individual | Traumatic experiences | 98 (98%) | 100 | 98 (98%) | - | - | - | 2 (2%) |
|  | Gender | 55 (62%) | 89 | 46 (52%) | - | 9 (10%) | - | 34 (38%) |
|  | Being female |  |  | 43 (48%) | - | 4 (4%) | - | - |
|  | Being male |  |  | 2 (2%) | 1 (1%) | 3 (3%) | - | - |
|  | Age (being older) | 35 (44%) | 80 | 28 (35%) | - | 7 (9%) | - | 45 (56%) |
|  | Chronic mental health issues | 19 (100%) | 19 | 18 (95%) | - | - | 1 (5%) | - |
|  | Chronic health issues | 18 (100%) | 18 | 18 (100%) | - | - | - | - |
|  | Language difficulty | 19 (70%) | 27 | 19 (70%) | - | - | - | 8 (30%) |
|  | Low SES | 13 (87%) | 15 | 9 (60%) | - | 3 (20%) | 1 (7%) | 2 (13%) |
|  | Coping strategy | 16 (80%) | 20 | 10 (50%) | - | 6 (30%) | - | 4 (20%) |
|  | Positive coping |  |  | 2 (10%) | - | 6 (30%) | - | - |
|  | Maladaptive coping |  |  | 8 (40%) | - | - | - | - |
|  | Higher Education | 21 (40%) | 52 | 3 (6%) | 3 (6%) | 15 (29%) | - | 31 (60%) |
|  | Religiosity | 6 (75%) | 8 | 1 (13%) |  | 5 (63%) |  | 2 (25%) |
|  | Self-efficacy | 4 (57%) | 7 | - | 2 (29%) | 2 (29%) |  | 3 (43%) |
| Family | Living conditions | 9 (100%) | 9 | 9 (100%) | - | - | - | - |
|  | Financial strain | 5 (100%) | 5 | 5 (100%) | - | - | - | - |
|  | Health or trauma experienced by loved ones | 4 (100%) | 4 | 4 (100%) | - | - | - | - |
|  | Social support | 27 (90%) | 30 | 1 (3%) | 2 (7%) | 24 (80%) | - | 3 (10%) |
|  | Marital status (being married) | 17 (50%) | 34 | 5 (15%) | - | 12 (35%) | - | 17 (50%) |
| Community | Social isolations | 4 (100%) | 4 | 4 (100%) | - | - | - | - |
|  | Social relations | 6 (100%) | 6 | - | 1 (17%) | 5 (83%) | - | - |
| Society | Postmigration stress | 36 (100%) | 36 | 36 (100%) | - | - | - | - |
|  | Unemployment | 19 (63%) | 30 | 17 (57%) | - | 1 (3%) | 1 (3%) | 11 (37%) |
|  | Perceived discrimination | 14 (93%) | 15 | 14 (93%) | - | - | - | 1 (7%) |
|  | Non-permanent legal status | 12 (92%) | 13 | 12 (92%) | - | - | - | 1 (8%) |
|  | Cultural identity | 11 (79%) | 14 | 5 (36%) | - | 6 (43%) | - | 3 (21%) |
|  | Integration/acculturation | 9 (82%) | 11 | - | - | 9 (82%) | - | 2 (18%) |
|  | Length of stay in the host country | 9 (100%) | 9 | 6 (67%) | - | 3 (33%) | - | - |
|  | Length of stay in refugee camps | 7 (100%) | 7 | 7 (100%) | - | - | - | - |

**Fig1. S3**

*Publication Years of Included Papers from 1984 to 2022*


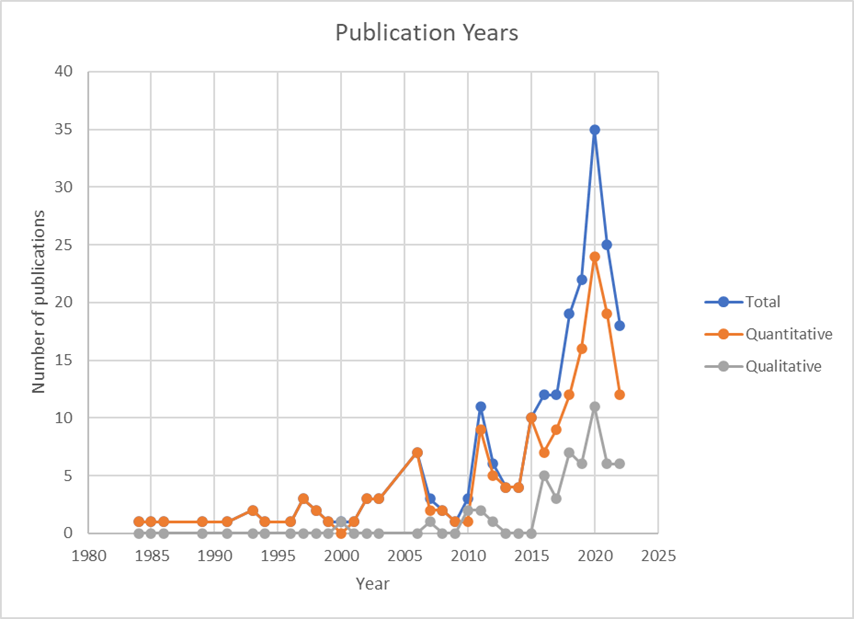


**Fig2. S3**

*Risk and Protective Factors influencing refugees’ resilience. Results based on a systematic review of 171 Quantitative Studies, presented in a Socio-Ecological Framework*


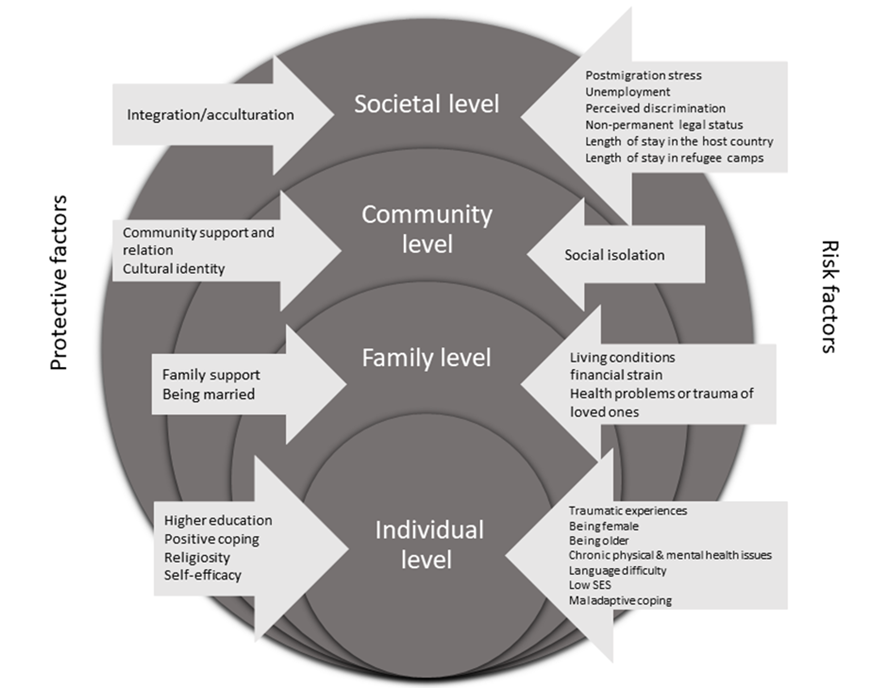


**Fig3. S3**

*Risk and Protective Factors influencing refugees’ resilience. Results based on a systematic review of 52 Qualitative Studies, presented in a Socio-Ecological Framework*


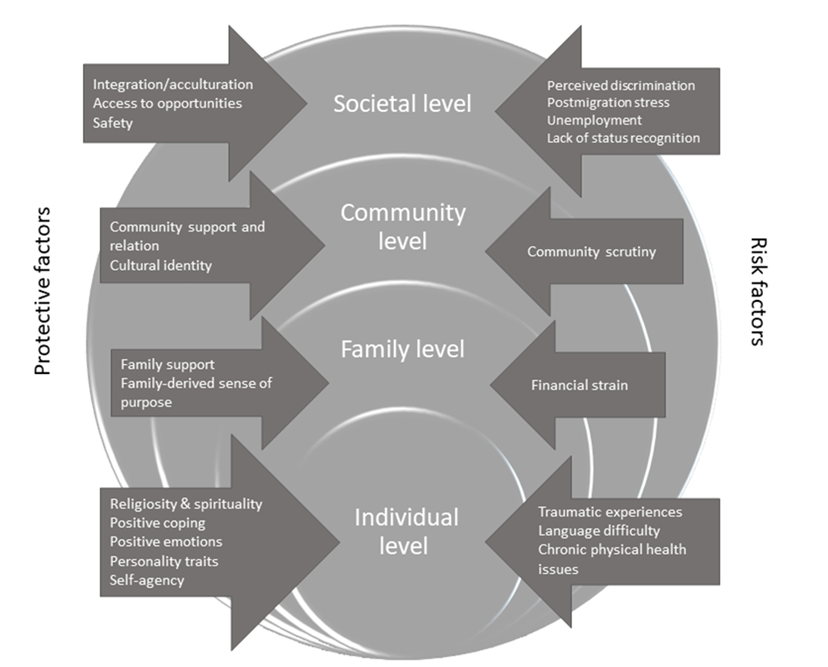


**Fig4. S3**

*Factor Clusters from Quantitative Studies Synthesis Based on the Frequency and Consistency of the Results*


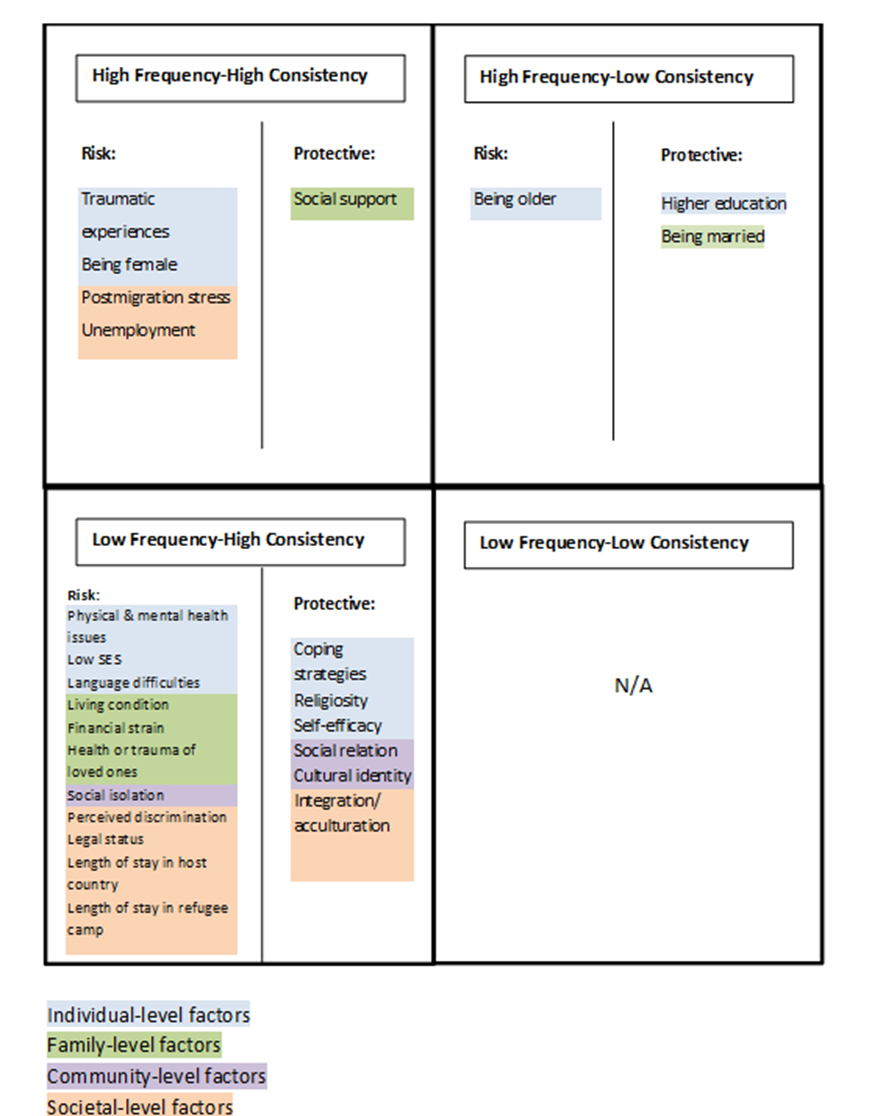

Supplement: sj-docx-3-tva-10.1177_15248380241284594 – Supplemental material for The Socio-Ecological Factors Associated with Mental Health Problems and Resilience in Refugees: A Systematic Scoping Review [file sj-docx-3-tva-10.1177_15248380241284594.docx]
