# Supplementary material for: The Socio-Ecological Factors Associated with Mental Health Problems and Resilience in Refugees: A Systematic Scoping Review
Source: Trauma Violence Abuse. 2024 Oct 8;26(3):598–616. doi: 10.1177/15248380241284594 (PMC12145474; doi:10.1177/15248380241284594)
Supplement: sj-docx-4-tva-10.1177_15248380241284594 – Supplemental material for The Socio-Ecological Factors Associated with Mental Health Problems and Resilience in Refugees: A Systematic Scoping Review [file sj-docx-4-tva-10.1177_15248380241284594.docx]

**Supplemental Material 4: Results Summary of Individual Quantitative Studies**

| Authors | Outcome Variables | Outcomes variables measurement | Samples | Sample Size | Protective Factors | Risk Factors |
| --- | --- | --- | --- | --- | --- | --- |
| Acarturk et al. - 2018 - Prevalence and predictors of posttraum | PTSD | Impact of Event Scale–Revised | Syrian refugees residing in a refugee camp in Turkey | 781 |  | Gender (being female) |
|  | Depression | Beck Depression Inventory |  |  |  | Chronic mental health issues |
|  |  |  |  |  |  | Health or trauma experienced of loved ones |
|  |  |  |  |  |  | Traumatic experiences |
|  |  |  |  |  |  | Time spent and experience at the camp |
| Acarturk et al. - 2020 - Prevalence and predictors of common me | Depression & Anxiety | Hopkins Symptoms Checklist | Syrian refugees living in Turkey | 1678 | SES | Gender (being female) |
|  | PTSD | PTSD Checklist |  |  |  | Chronic health issues |
|  |  |  |  |  |  | Chronic mental health issues |
|  |  |  |  |  |  | Problems accessing services |
|  |  |  |  |  |  | Unsafe environment |
|  |  |  |  |  |  | Police/law problems |
|  |  |  |  |  |  | No social or emotional support |
|  |  |  |  |  |  | Traumatic experiences |
| Ahmad et al. - 2020 - Depression-level symptoms among Syrian | Depression | Patient Health Questionnaire | Syrian refugees in Canada | 1924 | Location of data collection | Chronic mental health issues |
|  |  |  |  |  |  | Sponsorship type |
|  |  |  |  |  |  | Language difficulty |
|  |  |  |  |  |  | Living conditions |
|  |  |  |  |  |  | Lower perceived of control |
|  |  |  |  |  |  | Years of living in host country |
|  |  |  |  |  |  | Problems accessing services |
|  |  |  |  |  |  | No social or emotional support |
| Ahmad, Othman, Lou - 2020 - Posttraumatic Stress Disorder, Soci | PTSD | Harvard Trauma Questionnaire | Afghan refugees who visited a community health centre in Toronto. | 49 | Social support | Age (being older) |
|  |  |  |  |  |  | Unemployment |
|  |  |  |  |  |  | Chronic health issues |
| Ai, Peterson, Ubelhor - 2002 - War-related trauma and symptoms | PTSD | PTSD Symptom Scale | Kosovar refugees resettled in the US | 129 |  | Traumatic experiences |
|  |  |  |  |  |  | Gender (being female) |
|  |  |  |  |  |  | Marital status |
| Alduraidi, Dardas, Price - 2020 - Social Determinants of Resili | Resilience | Connor-Davidson Resilience Scale | Syrian refugees in Jordan | 151 | Living place |  |
|  |  |  |  |  | Education |  |
|  |  |  |  |  | Employment |  |
|  |  |  |  |  | SES |  |
| Alemi, James, Montgomery - 2015 - Correlates and Predictors of | Psychological distress | Afghan Symptom Checklist | Afghan refugees in San Diego | 130 | Education | Gender (being female) |
|  |  |  |  |  | Social support | Marital status |
|  |  |  |  |  |  | Financial strain |
|  |  |  |  |  |  | Unemployment |
|  |  |  |  |  |  | Postmigration stress |
|  |  |  |  |  |  | Age (being older) |
|  |  |  |  |  |  | Language difficulty |
| Alemi, Stempel - 2018 - Discrimination and distress among Afgha | Psychological distress | Talbieh Brief Distress Inventory | Afghan refugees in California, USA | 259 | Gender (being male) | Age (being older) |
|  |  |  |  |  | Education | Strong intra ethnic identity |
|  |  |  |  |  | Employment | Separation ethnic orientation |
|  |  |  |  |  | Ethnic identity | Perceived discrimination |
|  |  |  |  |  | English ability | Traumatic experiences |
|  |  |  |  |  | Social support |  |
| Alpak et al. - 2015 - Post-traumatic stress disorder among Syri | PTSD | PTSD Checklist | Syrian refugees in Turkey | 352 |  | Gender (being female) |
|  |  |  |  |  |  | Chronic mental health issues |
|  |  |  |  |  |  | Health or trauma experienced of loved ones |
|  |  |  |  |  |  | Traumatic experiences |
| Arfken et al. - 2018 - Recent Iraqi refugees Association betwee | PTSD | PTSD Checklist | Iraqi refugees in the US | 52 |  | Ethnicity |
|  | Depression & Anxiety | Hopkins Symptoms Checklist |  |  |  |  |
|  |  |  |  |  |  |  |
| Arnetz-2013-Resilience-as-a-protective-factor-a | PTSD | PTSD Checklist | Iraqi refugees residing in the Michigan | 75 |  | Gender (being female) |
|  |  |  |  |  |  | Traumatic experiences |
|  |  |  |  |  |  |  |
| Beiser et al. - 2011 - Stresses of Passage, Balms of Resettleme | PTSD | PTSD Symptom Scale | Sri Lankan Tamils in Toronto | 1603 | Satisfaction with life | Gender (being female) |
|  |  |  |  |  |  | Traumatic experiences |
|  |  |  |  |  |  | Discrimination |
|  |  |  |  |  |  | Poverty |
| Beiser, Hou - 2006 - Ethnic identity, resettlement stress and d | Depression | Depressive Affect Measure | Southeast Asian refugees in Canada | 647 | Age | Discrimination |
|  |  |  |  |  | Marital status | Unemployment |
|  |  |  |  |  | Ethnic identity | Language difficulty |
| Beiser, Johnson, Turner - 1993 - Unemployment, underemployment | Depression | Depressive Affect Measure | Southeast Asian refugees resettling in Vancouver, British Columbia | 1348 | Marital status | Chronic mental health issues |
|  |  |  |  |  | Education | Unemployment |
| Benson et al. - 2012 - Religious coping and acculturation stres | Acculturation stress | Social, Attitudinal, Familial and Environmental Acculturation Stress Scale | Bhutanese refugees in the southwestern US | 112 | English proficiency | Satisfaction of social support |
|  |  |  |  |  | Higher education | Age |
|  |  |  |  |  | Employment | Marital status |
|  |  |  |  |  |  | Religious coping |
| Bentley et al. - 2011 - The indirect effect of somatic complain | Depression & Anxiety | Hopkins Symptom Checklist | Somali refugees in Seattle | 74 |  | Traumatic experiences |
|  | PTSD | Harvard Trauma Questionnaire |  |  |  | Chronic health issues |
|  |  |  |  |  |  |  |
| Bentley et al. - 2012 - Post-Migration Stress as a Moderator Be | PTSD | Harvard Trauma Questionnaire | Somali refugees in Seattle | 74 |  | Traumatic experiences |
|  | Depression & Anxiety | Hopkins Symptom Checklist |  |  |  | Postmigration stress |
|  |  |  |  |  |  |  |
| Bentley, Ahmad, Thoburn - 2014 - Religiosity and posttraumatic | PTSD | Harvard Trauma Questionnaire | East African refugees living in the US | 59 | Religiosity | Traumatic experiences |
|  |  |  |  |  |  |  |
| Birman, Tran - 2008 - Psychological Distress and Adjustment of | Depression & Anxiety | Hopkins Symptom Checklist | Vietnamese refugees in the US | 212 | Social support | Gender (Being female) |
|  |  |  |  |  |  | Traumatic experiences |
|  |  |  |  |  |  | Strong intra ethnic identity |
| Böge et al (1). - 2020 - On Perceived Stress and Social Support Dep | PTSD | Harvard Trauma Questionnaire | Syrian refugees in Germany | 49 | Social support | Postmigration stress |
|  | Depression | Patient Health Questionnaire |  |  |  | Postmigration stress |
|  | Anxiety | Generalized Anxiety Disorder |  |  |  |  |
| Böge et al (2). - 2020 - On Perceived Stress and Social Support Dep | PTSD | Harvard Trauma Questionnaire | Syrian refugees in Jordan | 40 |  | Postmigration stress |
|  | Depression | Patient Health Questionnaire |  |  |  |  |
|  | Anxiety | Generalized Anxiety Disorder |  |  |  |  |
| Borho et al. - 2020 - The prevalence and risk factors for menta | PTSD | Essen Trauma Inventory | Syrian refugees living in Germany | 518 |  | Traumatic experiences |
|  | Depression | Patient Health Questionnaire |  |  |  | Discrimination |
|  | Anxiety | Generalized Anxiety Disorder Scale |  |  |  | Gender (being female) |
|  |  |  |  |  |  | Legal status |
| Brooks et al. - 2020 - Mental health of clinic-attending Syrian | Depression | Center for Epidemiological Studies Depression Scale | Syrian refugee women in Jordan | 507 | Marital status (being married) | Age (being older) |
|  | Anxiety | Generalized Anxiety Disorder |  |  |  | Intimate partner violence |
|  | PTSD | PTSD Checklist |  |  |  | Postmigration stress |
| Carlson, Rosser-Hogan - 1991 - Trauma experiences, posttraumati | PTSD | PTSD checklist | Cambodian refugees resettled in the US | 50 |  | Traumatic experiences |
|  | Depression & Anxiety | Hopkins Symptom Checklist |  |  |  |  |
| Carlsson, Mortensen, Kastrup - 2006 - Predictors of mental heal | PTSD | Harvard Trauma Questionnaire | Refugees (Iran, Afghan, Iraq, and others) in Denmark | 93 | Education | Traumatic experiences |
|  | Depression | Hamilton Depression Scale |  |  | Social relations |  |
|  | Anxiety | Hopkins Symptoms Checklist |  |  |  |  |
| Çelebi, Verkuyten, Bagci - 2017 - Ethnic identification, discri | Depression & Anxiety | Hopkins Symptom Checklist | Syrian refugees in Turkey. | 361 | Ethnic identity | Discrimination |
|  |  |  |  |  | Education |  |
| Cengiz et al. - 2019 - Posttraumatic stress disorder, posttraum | Resilience | Connor and Davidson Resilience Scale | Syrian refugees in Turkey | 310 | Social relations |  |
|  |  |  |  |  | Spirituality |  |
|  |  |  |  |  | Hope and positive outlook |  |
|  |  |  |  |  | Personality trait |  |
| Cheung - 1994 - Posttraumatic stress disorder among cambodian r | PTSD | Diagnostic Interview Schedule | Cambodian refugees in New Zealand. | 223 | Coping strategies | Age (Being older) |
|  |  |  |  |  |  | Traumatic experiences |
|  |  |  |  |  |  | Postmigration stress |
| Chung et al. - 2018 - Posttraumatic stress among Syrian r | PTSD | Harvard Trauma Questionnaire | Syrian refugees resettled in Sweden | 564 |  | Traumatic experiences |
| Chung et al. - 2020 - Posttraumatic stress disorder and psychia | PTSD | Harvard Trauma Questionnaire | Syrian refugees from Turkey and Sweden | 1197 |  | Traumatic experiences |
| Chung, Bemak, Kagawa-Singer - 1998 - Gender differences in psyc | Depression & Anxiety | Health Opinion Survey | Southeast Asian women refugees in California | 959 |  | Gender (Being female) |
|  |  |  |  |  |  | Language difficulty |
| Chung, Kagawa-Singer - 1993 (1) - Predictors of psychological distr | Depression & Anxiety | Health Opinion Survey | Vietnamese refugees in California | 867 |  | Traumatic experiences |
|  |  |  |  |  |  | Education |
|  |  |  |  |  |  | Time spent and experience at the camp |
|  |  |  |  |  |  | SES |
|  |  |  |  |  |  | Language difficulty |
|  |  |  |  |  |  | Living conditions |
|  |  |  |  |  |  | Gender (being female) |
|  |  |  |  |  |  | Age (Being older) |
| Chung, Kagawa-Singer - 1993 (2) - Predictors of psychological distr | Depression & Anxiety | Health Opinion Survey | Cambodian refugees in California | 590 |  | Age (being older) |
|  |  |  |  |  |  | Traumatic experiences |
|  |  |  |  |  |  | Time spent and experience at the camp |
|  |  |  |  |  |  | Language difficulty |
| Chung, Kagawa-Singer - 1993 (3) - Predictors of psychological distr | Depression & Anxiety | Health Opinion Survey | Lao refugees in California | 723 |  | Receiving public assistance |
|  |  |  |  |  |  | Unemployment |
|  |  |  |  |  |  | Years of living in host country |
|  |  |  |  |  |  | SES |
|  |  |  |  |  |  | Gender (Being female) |
|  |  |  |  |  |  | Traumatic experiences |
| Chung, Shakra - 2020 - The association between trauma centralit | PTSD | Harvard Trauma Questionnaire | Syrian refugees in Sweden | 475 | Self-efficacy | Traumatic experiences |
| Cummings et al. - 2011 - Correlates of depression among older K | Depression | Geriatric Depression Scale | Kurdish refugees in the US | 70 | SES | Age |
|  |  |  |  |  | Social support | Migratory grief |
|  |  |  |  |  |  | Health or trauma experienced of loved ones |
|  |  |  |  |  |  | Chronic health issues |
|  |  |  |  |  |  | Language difficulty |
| Demir et al. - 2020 - The role of emotion regulation as a media | PTSD | Harvard Trauma Questionnaire | Syrian refugees in Germany and Jordan | 89 |  | Maladaptive coping |
|  | Depression | Patient Health Questionnaire |  |  |  | Chronic mental health issues |
|  | Anxiety | Generalized Anxiety Disorder |  |  |  |  |
| Denkinger_2021_Longitudinal Changes in PTSD | PTSD | Impact of Event Scale | Yazidi Female Refugees with mean age 32.2 years in Germany | 116 | Religiosity |  |
|  |  |  |  |  | Social relations |  |
|  |  |  |  |  |  | Time spent and experience at the camp |
| Familiar et al. - 2021 - Conflict-related violence and menta | PTSD & Depression | Patient Health Questionnaire | Congolese refugee women in Uganda | 580 |  | Traumatic experiences |
| Faran - 2022 - Perceived Discrimination as a Moderator bet | Psychological distress | Brief Symptom Inventory | Darfuri asylum seekers in Israel | 300 |  | Postmigration stress |
|  | PTSD | PTSD Checklist |  |  |  | Discrimination |
| Feyera et al. - 2015 - Prevalence of depression and associated | Depression | Patient Health Questionnaire | Somali refugees in Ethiopia | 847 |  | Gender (Being female) |
|  |  |  |  |  |  | Marital status |
|  |  |  |  |  |  | Traumatic experiences |
|  |  |  |  |  |  | Trauma experienced by loved ones |
|  |  |  |  |  |  | Living conditions |
| Frounfelker -2022 -Past trauma resettlement stress and | Anxiety | Generalized Anxiety Disorder | Bhutanese in the US and Canada | 190 |  | Traumatic experiences |
|  | Depression | Patient Health Questionnaire |  |  |  | Postmigration stress |
| Gautam et al. - 2021 - Psychological distress among Bhutane | Depression & Anxiety | Depression Anxiety Stress Scale | Bhutanese refugees in the United States | 376 |  | Social integration deficit |
|  |  |  |  |  |  | Social isolation |
|  |  |  |  |  |  | Chronic health issues |
|  |  |  |  |  |  | Years of living in host country |
|  |  |  |  |  |  | Employment |
| Georgiadou et al. - 2018 - Prevalence of mental distress among | PTSD | Essen Trauma Inventory | Syrian refugees living in Germany | 518 |  | Age (being older) |
|  | Depression | Patient Health Questionnaire |  |  |  | Age (being younger) |
|  | Anxiety | Generalized Anxiety Disorder |  |  |  | Legal status |
|  |  |  |  |  |  | Traumatic experiences |
|  |  |  |  |  |  | Chronic mental health issues |
|  |  |  |  |  |  | Gender (Being female) |
| Gerritsen et al. - 2006 - Physical and mental health of Afghan, | Depression & Anxiety | Hopkins Symptoms Checklist | Refugees and asylum seekers from Afghanistan, Iran, and Somalia resettled in the Netherlands | 410 |  | Gender (being female) |
|  | PTSD | Harvard Trauma Questionnaire |  |  |  | Traumatic experiences |
|  |  |  |  |  |  | No social or emotional support |
|  |  |  |  |  |  | Postmigration stress |
|  |  |  |  |  |  | Legal status |
| Getnet, Medhin, Alem - 2019 - Symptoms of post-traumatic str | Depression | Center for Epidemiological Studies Depression Scale | Eritrean refugees in Ethiopia | 562 | Sense of coherence | Postmigration stress |
|  | PTSD | Primary Care PTSD Screener |  |  | Coping strategies | Traumatic experiences |
|  |  |  |  |  | Social support | Coping strategy (emotion-oriented) |
|  |  |  |  |  |  | Maladaptive coping |
|  |  |  |  |  |  | Gender (being female) |
| Ginesini - 2018 - Trauma, Faith, and Resilience | Resilience | Wagnild & Young Resilience scale | Female refugees from various countries in Italy | 18 | Positive emotion | Chronic mental health issues |
| Haldane, Nickerson - 2016 - The impact of interpersonal and non | PTSD | PTSD Symptom Scale Interview | Refugees and asylum seekers from various countries living in Australia | 91 |  | Traumatic experiences |
|  | Depression & Anxiety | Hopkins Symptoms Checklist |  |  |  | Gender (being female) |
| Hamrah et al. - 2020 - Occurrence and correlates of depressive | Depression | Hopkins Symptoms Checklist | Afghan refugees resettled in Australia | 66 | Job status in country of origin | Gender |
|  |  |  |  |  |  | Social Isolation |
|  |  |  |  |  |  | physical inactivity |
| Hamrah et al. - 2020 - The prevalence and correlates of symptom | PTSD | Impact of Event Scale-Revised | Afghan refugees resettled in Australia | 66 |  | Language difficulty |
|  |  |  |  |  |  | Traumatic experiences |
|  |  |  |  |  |  | Chronic mental health issues |
| Hinton et al.- 1997 - Predictors of depression among refugees | Depression | Hopkins Symptoms Checklist | Vietnamese refugees in the US | 114 |  | Chronic mental health issues |
|  |  |  |  |  |  | Age (being older) |
|  |  |  |  |  |  | Language difficulty |
|  |  |  |  |  |  | Ethnicity |
|  |  |  |  |  |  | Traumatic experiences |
| Hinton, Nickerson, Bryant - 2011 - Worry, worry attacks, and PT | PTSD | PTSD Checklist | Cambodian refugees in the US | 201 |  | Traumatic experiences |
| Hoffman et al. - 2018 - The relationship between moral injury a | PTSD | Posttraumatic Diagnostic Scale | Refugees from various countries in Australia | 222 |  | Violation of one's moral values |
|  | Depression | Patient Health Questionnaire |  |  |  | Traumatic experiences |
| Hooberman et al. - 2010 - Resilience in trauma-exposed refugees | PTSD | Harvard Trauma Questionnaire | Refugees from West Africa in New York city | 77 |  | Maladaptive coping |
|  |  |  |  |  |  | Social comparisons |
| Hossain et al. - 2020 - Disability, violence, and mental health | PTSD | Harvard Trauma Questionnaire | Somali refugees in Kenya | 209 |  | Traumatic experiences |
|  | Anxiety | Generalised anxiety disorder |  |  |  | Chronic health issues |
|  | Depression | Patient Health Questionnaire |  |  |  |  |
| Hossain_2021_Predisplacement abuse and postdisplacement fac | PTSD, depression, & anxiety | Impact of Event Scale | Rohingya refugees aged 18 years older in Bangladesh | 1184 | Employment | Traumatic experiences |
|  |  |  |  |  | Social support |  |
| Hussain, Bhushan - 2011 - Posttraumatic stress and growth among | PTSD | Impact of Event Scale | Tibetan refugees in India | 226 | Education | Traumatic experiences |
|  |  |  |  |  |  | Gender (being female) |
|  |  |  |  |  |  | Age (being older) |
|  |  |  |  |  |  | Marital status |
| Ichikawa, Nakahara, Wakai - 2006 - Effect of post-migration det | PTSD | Harvard Trauma Questionnaire | Afghan asylum seekers in Japan | 55 |  | Traumatic experiences |
|  | Depression & Anxiety | Hopkins Symptoms Checklist |  |  |  | Living conditions |
|  |  |  |  |  |  |  |
| Idemudia et al. - 2014 - Genfer differences in trauma and postt | PTSD | PTSD checklist | Zimbabwean refugees in South Africa | 125 |  | Chronic mental health issues |
|  |  |  |  |  |  | Gender (being female) |
|  |  |  |  |  |  | Traumatic experiences |
| Jamil et al. - 2007 - Mental health symptoms in Iraqi refugees | PTSD | Posttraumatic Stress Diagnostic Scale | Iraqi refugees in the US | 116 |  | Gender (being female) |
|  | Depression & Anxiety | Hopkins Symptoms Checklist |  |  |  |  |
| Jankovic-Rankovic et al. - 2022 - Transient refugees' social su | Psychological distress | Impact of Event Scale | Refugees from Afghanistan in Serbia | 76 | Social support |  |
| Javanbakht et al. - 2020 - Perceived health, adversity, and pos | PTSD | PTSD checklist | Syrian and Iraqi refugees in the US | 152 |  | Chronic mental health issues |
| Jeon, Eom, Min - 2013 - A 7-year follow-up study on the mental | PTSD | PTSD Symptom Scale | North Korean refugees in South Korea | 106 |  | Postmigration stress |
|  | Depression | Beck Depression Inventory |  |  |  | Traumatic experiences |
|  | Anxiety | Hopkins Symptoms Checklist |  |  |  | Chronic mental health issues |
| Jorden, Matheson, Anisman - 2009 - Supportive and unsupportive | Psychological distress | Acculturation Stress Scale | Somali refugees in Canada | 169 |  | Traumatic experiences |
|  | Depression | Beck Depression Inventory |  |  |  | Postmigration stress |
|  |  |  |  |  |  | Lack of social support |
| Jorgenson, Nilsson - 2021 - The Relationship Among Trauma, Accu | PTSD | PTSD Symptom Scale-Self Report | Somali refugees resettled in the US | 80 | Integration/acculturation | Traumatic experiences |
|  | Depression & Anxiety | Hopkins Symptoms Checklist |  |  | Language ability | Acquiring identity of host country |
|  |  |  |  |  |  | Gender (being female) |
| Kahve et al. - 2020 - Evaluating the Relationship Between Po | PTSD | Clinician-Administered Post-Traumatic Stress Disorder Scale | Iraqi Turkoman refugees who migrated to Turkey | 101 |  | Chronic mental health issues |
| Kartal et al. - 2018 - Traumatic exposure, acculturative stress | PTSD | Posttraumatic Stress Diagnostic Scale | Bosnian refugees resettled in Australia and Austria | 138 | Integration/ acculturation | Traumatic experiences |
|  | Depression & Anxiety | Depression Anxiety Stress Scale |  |  |  | Postmigration stress |
|  |  |  |  |  |  |  |
| Kartal, Alkemade, Kiropoulos - 2019 - Trauma and mental health | PTSD | Posttraumatic Stress Diagnostic Scale | Bosnian refugees resettled in Australia and Austria | 138 | Language ability | Traumatic experiences |
|  | Depression & Anxiety | Depression Anxiety Stress Scale |  |  |  |  |
| Kartal, Kiropoulos - 2016 (1) - Effects of acculturative stress | Depression & Anxiety | Depression Anxiety Stress Scale | Bosnian refugees in Australia | 56 |  | Traumatic experiences |
| Kartal, Kiropoulos - 2016 (2) - Effects of acculturative stress | PTSD | Posttraumatic Stress Diagnostic Scale | Bosnian refugees in Austria | 82 |  | Postmigration stress |
|  | Depression & Anxiety | Depression Anxiety Stress Scale |  |  |  | Traumatic experiences |
| Kashyap, Page, Joscelyne - 2019 - Post-migration treatment targ | PTSD | Harvard Trauma Questionnaire | Refugees from various countries living in New York | 323 | Age | Traumatic experiences |
|  | Depression | Patient Health Questionnaire |  |  | Immigration status | Chronic health issues |
|  |  |  |  |  | Gender (female) |  |
|  |  |  |  |  | Living place |  |
| Kaya et al. - 2019 - Posttraumatic Stress and Depression among | PTSD | Harvard Trauma Questionnaire | Syrian refugees living in Turkey urban area | 420 |  | Gender (being female) |
|  | Depression | Beck Depression Inventory |  |  |  | Chronic mental health issues |
|  |  |  |  |  |  | Chronic health issues |
|  |  |  |  |  |  | Traumatic experiences |
|  |  |  |  |  |  | SES |
| Keller et al. - 2003 - Mental health of detained asylum seekers | PTSD | Harvard Trauma Questionnaire | Asylum seekers detained in  New York, New Jersey, and Pennsylvania. | 70 |  | Time spent and experience at the camp |
|  | Depression & Anxiety | Hopkins symptom checklist |  |  |  |  |
| Keller et al. - 2006 - Traumatic experiences and psychological | PTSD | Harvard Trauma Questionnaire | Refugees from various countries living in New York | 325 | Age | Gender (being female) |
|  | Depression & Anxiety | Hopkins symptom checklist |  |  | Legal status | Traumatic experiences |
| Khan, Haque - 2021 - Trauma, mental health, and everyday functi | PTSD | PTSD Checklist | Rohingya refugees in Malaysia | 100 |  | Traumatic experiences |
|  | Depression | Patient Health Questionnaire |  |  |  |  |
|  | Anxiety | Generalized Anxiety Disorder |  |  |  |  |
| Kim et al. - 2019 - Trauma, Discrimination, and Psychological D | Psychological distress | Kessler Psychological Distress Scale | Vietnamese refugees in the US | 291 |  | Gender (being female) |
|  |  |  |  |  |  | Age (being older) |
|  |  |  |  |  |  | Traumatic experiences |
| Kim et al. - 2019 - Pre-migration Trauma, Repatriation Experien | Psychological distress | Impact of Event Scale | North Korean refugees in South Korea | 698 | Gender (female) | Traumatic experiences |
|  |  |  |  |  | Age (being older) | Chronic health issues |
|  |  |  |  |  | Social support |  |
| Kim et al. -2022- Social determinants of mental health | Psychological distress | Refugee Health Screener | Karen Refugees in the United States | 201 | Education | Chronic health issues |
|  |  |  |  |  |  | Traumatic experiences |
|  |  |  |  |  |  | Religiosity |
| Kim, Yun, von Denkowski - 2022 - Violence Against North Korean | PTSD | Impact of Event Scale | North Korean Refugee Women in South Korea | 627 | Social support | Gender (being female) |
|  | Depression | Center for Epidemiological Study Depression |  |  |  | Traumatic experiences |
|  | Anxiety | Beck’s anxiety inventory |  |  |  | Age (being older) |
|  |  |  |  |  |  | SES (high) |
|  |  |  |  |  |  | Maladaptive coping |
| Kira et al. - 2017 - A Threatened Identity The Mental Health St | PTSD | Clinician-Administered PTSD Scale | Syrian refugees in Egypt | 196 |  | Traumatic experiences |
|  |  |  |  |  |  | Experience of identity threat |
| Kivling-Boden, Sundbom - 2001 - Life situation and posttraumati | PTSD | Harvard Trauma Questionnaire | Yugoslavian refugees in Sweden | 27 |  | Gender (being female) |
| Knipscheer et al. - 2015 - Trauma exposure and refugee status a | PTSD & Depression | Harvard Trauma Questionnaire | Refugees from the Middles East, Sub-Sahran Africa, and Balkan Europe in the Netherlands | 688 | Gender | Traumatic experiences |
|  |  |  |  |  |  | Legal status |
| Knipscheer, Kleber - 2006 - The relative contribution of posttr | Depression & Anxiety | Posttraumatic Reactions | Bosnian refugees in the Netherlands | 78 | Maintaining cultural identity | Traumatic experiences |
|  |  |  |  |  | Integration/acculturation |  |
| Kroll et al. - 1989 - Depression and posttraumatic stress disor | PTSD & Depression | Interview based on clinical diagnosed of DSM-III criteria | Southeast Asian refugees in the US | 404 |  | Marital status |
|  |  |  |  |  |  | Traumatic experiences |
| Kuittinen et al. - 2017 - Causal attributions of mental health | Depression | Beck Depression Inventory | Somali refugees in Finland | 128 | Religiosity |  |
| Kurt et al. -2021- Traumatic experiences, acculturation | Psychological distress & Depression | General Health Questionnaire | Syrian refugees in Turkey | 409 | Acculturation | Gender (being female) |
| Kuttikat & Jettner - 2014 - Migration stressors, psychological | Psychological distress | Symptoms Check List | Sri lankan Tamil refugees in India | 83 | Number of children |  |
|  |  |  |  |  |  | Traumatic experiences |
|  |  |  |  |  |  | Postmigration stress |
| Leaman, Gee - 2012 - Religious coping and risk factors for psyc | PTSD | Harvard Trauma Questionnaire | African refugees in the US | 131 |  | Traumatic experiences |
|  | Depression | Hopkins Symptom Checklist |  |  |  | Negative religious coping |
| Lee, Noh, et al. - 2021- The effects of inhuman treatment | PTSD | PTSD Checklist | North Korean refugees in South Korea | 300 |  | Traumatic experiences |
| Lenferink et al. -2022- Course and predictors of PTSD | PTSD | PTSD Scale | Refugees from Iraq, Syria, Iran, Afghanistan, Sri Lanka, Burma, and Pakistan in Australia | 613 |  | Discrimination |
|  |  | Patient Health Questionnaire |  |  |  | Traumatic experiences |
| Lie - 2002 - A 3-year follow-up study of psychosocial functioni | PTSD | Post-traumatic Symptom Scale | Refugees from various countries in Norway | 240 | Social relations | Traumatic experiences |
|  |  |  |  |  | Social support |  |
| Lillee, Thambiran, Laugharne - 2015 - Evaluating the mental hea | PTSD | PTSD treatment screener | Refugees from various countries in Australia | 300 |  | Marital status |
|  |  |  |  |  |  | Number of children |
| Lim, Han - 2016 - A Predictive Model on North Korean Refugees' | Resilience | Self-develop Resilience Scale | North Korean refugees in South Korea | 445 | Self-efficacy |  |
| Lin, Ihle, Tazuma - 1985 - Depression among vietnamese refugees | Depression | Vietnamese Depression Scale | Vietnamese refugees in the US | 92 |  | Age (being older) |
|  |  |  |  |  |  | Marital status |
|  |  |  |  |  |  | Language difficulty |
|  |  |  |  |  |  | Limited local experience and education |
| Lindencrona, Ekblad, Hauff - 2008 - Mental health of recently | PTSD | Core symptoms of post-traumatic stress | Middle Eastern refugees in Sweden | 124 | Self-agency | Traumatic experiences |
|  |  |  |  |  | Coping strategy |  |
|  |  |  |  |  | Gender (female) |  |
| Lor et al. - 2022 - Refugee-related trauma patterns and mental | PTSD | Harvard Trauma Questionnaire | Hmong refugees in the United  States | 219 |  | Traumatic experiences |
|  | Depression & Anxiety | Brief Symptom Inventory |  |  |  |  |
| Mahmood et al. - 2019 - Post-Traumatic stress disorder and depr | PTSD | PTSD Checklist | Syrian Kurdish refugees in Iraq | 988 |  | Gender (being female) |
|  | Depression | Hopkins Symptom Checklist |  |  |  | Age (being older) |
|  |  |  |  |  |  | Time spent and experience at the camp |
|  |  |  |  |  |  | Traumatic experiences |
|  |  |  |  |  |  | Growing up in urban area |
| Miller et al. - 2002 (1) - The relative contribution of war experie | PTSD | Posttraumatic Stress Scale | Clinical group of Bosnian refugees in the US | 59 | Coping strategy | Traumatic experiences |
|  | Depression | Center for Epidemiological Studies Depression Scale |  |  |  |  |
| Miller et al. - 2002 (2)- The relative contribution of war experie | PTSD | Posttraumatic Stress Scale | Nonclinical group of Bosnian refugees in the US | 40 |  | Traumatic experiences |
|  | Depression | Center for Epidemiological Studies Depression Scale |  |  |  | Social isolation |
| Minihan et al. - 2018 - Patterns and predictors of posttraumati | PTSD | Post-traumatic Diagnostic Scale | Refugees and asylum seekers from various countries in Australia | 246 |  | Traumatic experiences |
|  |  |  |  |  |  | Postmigration stress |
| Mollica, Caridad, Massagli - 2007 - Longitudinal study of postt | PTSD & Depression | Hopkins Symptom Checklist | Bosnian refugees in Croatia | 376 |  | Traumatic experiences |
|  |  |  |  |  |  |  |
| Mölsä et al. - 2017 - Mental health among older refugees the ro | PTSD | Trauma Screening Questionnaire | Somali refugees in Finland | 128 | Religiosity | Traumatic experiences |
|  | Depression | Beck’s Depression Inventory |  |  |  | Years of living in host country |
|  |  |  |  |  |  | Legal status |
|  |  |  |  |  |  | Discrimination |
| Momartin et al. - 2003 - Dimensions of trauma associated with p | PTSD | Clinician-administered PTSD scale | Bosnian refugees in Australia | 126 |  | Traumatic experiences |
| Morgan, Melluish, Welham - 2017 - Exploring the relationship be | PTSD | Harvard Trauma Questionnaire | Refugees from various countries in the UK | 97 |  | Social isolation |
|  |  | Hopkins Symptom Checklist |  |  |  | Legal status |
| Mwanamwambwa, Pillay - 2022 - Posttraumatic stress disorder and | PTSD | Impact of Event Scale | Rwandan refugees in Zambia. | 267 |  | Unemployment |
|  | Psychological distress | General Health Questionnaire |  |  |  | Financial strain |
|  |  |  |  |  |  | Education (low) |
|  |  |  |  |  |  | Living conditions |
|  |  |  |  |  |  | Marital status (being married) |
|  |  |  |  |  |  | Number of children (no children) |
| Naal et al. - 2021 - Prevalence of depression symptoms and asso | Depression | Patient Health Questionnaire | Syrian refuges in Lebanon | 3255 |  | Age (being older) |
|  |  |  |  |  |  | Gender (being female) |
|  |  |  |  |  |  | Marital status |
|  |  |  |  |  |  | Chronic health issues |
|  |  |  |  |  |  | Chronic mental health issues |
| Nakash et al. - 2015 - The association between acculturation pa | Depression & Anxiety | Hopkins Symptom Checklist | Eritrean and Sudanese asylum sekers in Israel | 118 |  | Traumatic experiences |
|  |  |  |  |  |  | Postmigration stress |
| Nam-2016-Family-functioning-resilience-and-d | Depression | Center for Epidemiologic Studies Depression | North Korean refugees in South Korea | 304 | Social support |  |
| Nesterko et al. - 2020 - Factors predicting symptoms of somatiz | Depression | Patient Health Questionnaire | Refugees from various countries in Germany | 502 |  | Postmigration stress |
|  | PTSD | PTSD Checklist |  |  |  | Chronic health issues |
|  | Anxiety | Hopkins Symptom Checklist-25 |  |  |  | Gender (being female) |
|  |  |  |  |  |  | Marital status |
|  |  |  |  |  |  | Traumatic experiences |
| Nicassio et al. - 1986 - Emigration stress and langltage profic | Depression | Center for Epidemiological Studies Depression Scale | Laotian refugees in the US | 48 | Language ability | Postmigration stress |
| Nicholson - 1997 - The influence of pre-emigration and postemig | PTSD | Harvard Trauma Questionnaire | Southeast Asian refugees in the US | 447 | SES | Postmigration stress |
|  | Depression & Anxiety | Hopkins Symptom Checklist |  |  |  | Chronic health issues |
|  |  |  |  |  |  | Traumatic experiences |
| Nickerson et al. - 2007 - Comorbidity of posttraumatic stress d | PTSD | Posttraumatic Diagnostic Scale | Refugees from various countries resettled in Switzerland | 134 |  | Traumatic experiences |
|  | Depression | Hopkins Symptom Checklist |  |  |  | Age (being younger) |
|  |  |  |  |  |  | Years of living in host country |
|  |  |  |  |  |  | Postmigration stress |
|  |  |  |  |  |  | Chronic mental health issues |
| Nickerson et al. - 2011 - The familial influence of loss and tr | PTSD | Harvard Trauma Questionnaire | Iraqi refugees residing in Australia | 315 |  | Maladaptive coping |
|  | Depression | Hopkins Symptom Checklist |  |  |  | Traumatic experiences |
| Nilsson et al. - 2021 - Physical activity, post-traumatic st | PTSD | Harvard Trauma Questionnaire | Asylum seekers from Afghanistan, Eritrea, Iraq, Somalia, and Syria in Sweden | 455 | Physical activity | Traumatic experiences |
|  |  |  |  |  | Gender (being female) |  |
| Noh et al. - 1999 - Perceived Racial Discrimination , Depressio | Depression | Depression Scale | Southeast Asian refugees in Canada | 647 |  | Discrimination |
|  |  |  |  |  | Coping strategy |  |
|  |  |  |  |  | Age |  |
|  |  |  |  |  | Education |  |
|  |  |  |  |  | Employment |  |
| Nosè et al. - 2018 - Prevalence and Correlates of Psychological | PTSD | Life Events Checklist | Asylum seekers and refugees from various countries in Italy | 109 | Years of living in host country | Traumatic experiences |
|  | Depression | Hamilton Rating Scale for Depression |  |  | Time after departure from origin country |  |
|  |  |  |  |  | Marital status |  |
| Pak, Yurtbakan, Acarturk - 2022 - Social Support and Resilience | Resilience | Connor-Davidson Resilience Scale | Syrian refugees in Turkey | 339 | Gender (being male) |  |
|  |  |  |  |  | Education (high) |  |
|  |  |  |  |  | Social support |  |
|  |  |  |  |  | Self-efficacy |  |
| Papastamatelou, Unger, Zachariadis - 2021 - Time perspectives | PTSD | Posttraumatic Symptom Scale | Syrian refugees in Greece | 77 |  | Time perspectives |
| Poudel-Tandukar et al. - 2020 - Coping strategies and stress am | PTSD | Cohen Perceived Stress Scale | Bhutanese refugees in the US | 225 | Coping strategy |  |
| Poudel-tandukar-2019-Resilience-and-anxiety-or-depressio | Depression & Anxiety | Hopkins Symptom Checklist | Bhutanese refugees in the US | 225 | Personality trait |  |
| Regev, Slonim-Nevo - 2019 - Trauma and mental health in Darfuri | PTSD | PTSD Checklist | Darfuri (African) refugees living in Israel | 300 |  | Traumatic experiences |
|  | Depression | Brief Symptom Inventory |  |  |  |  |
| Renner et al. - 2021 - Predictors of psychological distress in | Anxiety | Generalized Anxiety Disorder | Syrian refugees in Germany | 133 | Social Support | SES (low) |
|  | Depression | Patient Health Questionnaire |  |  |  | Mental health stigma |
|  | Anxiety | Posttraumatic Diagnostic Scale |  |  |  | Gender (being female) |
|  |  |  |  |  |  | Education (high) |
|  |  |  |  |  |  | Connection to home country |
|  |  |  |  |  |  | Traumatic experiences |
| Renner, Laireiter, Maier - 2012 - Social support as a moderator | Depression & Anxiety | Hopkins Symptom Checklist | Refugees from multiple countries in Austria | 63 | Social support from sponsors |  |
|  | PTSD | Harvard Trauma Questionnaire |  |  |  |  |
| Riley et al. - 2017 - Daily stressors, trauma exposure, and men | PTSD | Harvard Trauma Questionnaire | Rohingya refugees in Bangladesh | 148 |  | Traumatic experiences |
|  | Depression | Hopkins Symptom Checklist |  |  |  | Age (being older) |
|  |  |  |  |  |  | Gender (being female) |
|  |  |  |  |  |  | Postmigration stress |
| Saab et al. - 2020 - Psychological Distress among Syrian Ref | Psychological distress | General Health Questionnaire | Syrian refugees in Lebanon | 35 | SES |  |
| Sagaltici, Gokay, Altindag - 2020 - Traumatic Life Events and S | PTSD | Clinician-Administered PTSD Scale | Syrian refugees in Turkey | 342 |  | Traumatic experiences |
|  |  |  |  |  |  | Gender (being female) |
|  |  |  |  |  |  | Age |
| Salhi et al. - 2010 - The relationship of pre- and post-resettl | PTSD | Harvard Trauma Questionnaire | Somali refugees in the US and Canada | 383 |  | Traumatic experiences |
|  | Depression | Hopkins Symptom Checklist |  |  |  | Police/law problems |
|  |  |  |  |  |  |  |
| Sangalang et al. - 2019 (1) - Trauma, Post-Migration Stress, and Me | Depression & Anxiety | Kessler Psychological Distress scale | Asian refugees in the US | 354 |  | Discrimination |
|  |  |  |  |  |  | Postmigration stress |
|  |  |  |  |  |  | Family conflict |
|  |  |  |  |  |  | Traumatic experiences |
| Sangalang et al. - 2019 (2) - Trauma, Post-Migration Stress, and Me | Depression & Anxiety | Kessler Psychological Distress scale | Latinos refugees in the US | 306 |  | Postmigration stress |
|  |  |  |  |  |  | Family conflict |
| Schlaudt et al. - 2020 - Traumatic Experiences and Mental He | PTSD, depression, & anxiety | Refugee Health Screener | Refugees from diverse nationalities in the US | 8149 |  | Traumatic experiences |
|  |  |  |  |  |  | Age |
|  |  |  |  |  |  | Gender (being female) |
| Schweitzer et al. - 2011 - Mental health of newly arrived Burme | Depression & Anxiety | Hopkins Symptom Checklist | Burmese refugees in Australia | 70 |  | Postmigration stress |
|  |  |  |  |  |  |  |
|  |  |  |  |  |  |  |
| Sengoelge et al. - 2019 (1)- Identifying subgroups of refugees fro | PTSD | Harvard Trauma Questionnaire | "Class 1" Syrian refugees resettled in Sweden | 546 |  | Gender (being female) |
|  | Depression & Anxiety | Hopkins Symptom Checklist |  |  |  | Living conditions |
|  |  |  |  |  |  | Age (being older) |
| Sengoelge et al. - 2019 (2)- Identifying subgroups of refugees fro | PTSD | Harvard Trauma Questionnaire | "Class 2" Syrian refugees resettled in Sweden | 569 |  | Age (being older) |
|  | Depression & Anxiety | Hopkins Symptom Checklist |  |  |  | Gender (being female) |
|  |  |  |  |  |  | Education |
|  |  |  |  |  |  | Living conditions |
| Sengoelge et al. - 2019 (3)- Identifying subgroups of refugees fro | PTSD | Harvard Trauma Questionnaire | "Class 3" Syrian refugees resettled in Sweden | 281 |  | Age (being older) |
|  | Depression & Anxiety | Hopkins Symptom Checklist |  |  |  | Living conditions |
|  |  |  |  |  |  |  |
| Sengoelge et al. - 2020 - Exploring Social and Financial Har | Depression & Anxiety | Hopkins Symptom Checklist | Asylum seekers from various countries in Sweden | 455 | Social support | Gender (being female) |
|  |  |  |  |  |  | Financial strain |
| Shannon et al. - 2015 - Torture, War Trauma, and Mental Health | PTSD & Depression | Self-develop Distress Screener | Karen refugees in the US | 179 | Education | Traumatic experiences |
|  |  |  |  |  |  | Gender (being female) |
| Shaw et al. - 2019 - Emotional distress among Rohingya refugees | Psychological distress | Refugee Health Screener | Rohingya refugees in Malaysia | 115 |  | Age (being older) |
| Shin, Yoon - 2018 - Acculturative stress as a mental health pre | Depression & Anxiety | Hopkins Symptom Checklist | North Korean refugees residing in South Korea | 1200 | Social support | Postmigration stress |
|  |  |  |  |  | Job status/employment |  |
| Silove et al. - 1997 - Anxiety, depression and PTSD in asylum s | Depression & Anxiety | Hopkins Symptom Checklist | Refugees from various countries living in Australia | 40 |  | Conflict with immigration officials |
|  | PTSD | The Composite International Diagnostic  Interview |  |  |  | Gender (being female) |
|  |  |  |  |  |  | SES |
|  |  |  |  |  |  | Loneliness |
|  |  |  |  |  |  | Traumatic experiences |
|  |  |  |  |  |  | Dealing with complicated paperwork |
|  |  |  |  |  |  | Unemployment |
|  |  |  |  |  |  | Discrimination |
| Silove et al. - 1998 - Trauma exposure, postmigration stressors | PTSD, depression, & anxiety | Harvard Trauma Questionnaire | Sri Lankan Tamil asylum seekers in Australia | 62 |  | Postmigration stress |
|  |  |  |  |  |  |  |
| Sim, Bowes, Gardner - 2019 - The Promotive Effects of Social | Resilience | Depression Anxiety and Stress Scale | Syrian refugees in Lebanon | 292 | Social support |  |
|  |  |  |  |  | Education |  |
| Smeekes et al. - 2017 - Social identity continuity and mental h | Depression & Anxiety | Hopkins Symptom Checklist | Syrian refugees in Turkey. | 361 | Maintaining cultural identity | Gender (being female) |
|  |  |  |  |  | Education |  |
|  |  |  |  |  | Location of data collection |  |
| Spiller et al. - 2019 - Emotional reactivity, emotion regulatio | PTSD | PTSD Symptom Scale | Refugees from multiple countries in Australia | 81 |  | Chronic mental health issues |
|  |  |  |  |  | Emotion regulation capacity |  |
| Starck et al. - 2020 - The relationship of acculturation, traum | Depression | Hopkins Symptom Checklist | Female refugees from various countries in Germany | 98 | Integration/acculturation | Traumatic experiences |
|  |  |  |  |  |  |  |
| Steel et al. - 2006 - Impact of immigration detention and tempo | PTSD | Harvard Trauma Questionnaire | Middle East refugees in Australia | 241 |  | Legal status |
|  | Depression | Hopkins Symptom Checklist |  |  |  | Time spent and experience at the camp |
|  |  |  |  |  |  | Living conditions |
| Steel et al. - 2011 - Two year psychosocial and mental health o | PTSD | Harvard Trauma Questionnaire | Refugees from Iran and Afghanistan in Australia | 104 | Immigration status | Postmigration stress |
|  | Depression & Anxiety | Hopkins Symptom Checklist |  |  |  |  |
| Steel et al. - 2016 - The Psychological Consequences of Pre-Emi | Depression & Anxiety | Hopkins Symptom Checklist | African refugees residing in Sweden | 420 |  | Gender (being female) |
|  | PTSD | Harvard Trauma Questionnaire |  |  |  | Traumatic experiences |
|  |  |  |  |  |  | Postmigration stress |
|  |  |  |  |  |  | Education |
| Stempel-2016-Gendered-sources-of-distress-and-re | Psychological distress | Talbieh Brief Distress Inventory | Afghan refugees in the US | 259 | Age (being younger) | Discrimination |
|  |  |  |  |  | Education | Postmigration stress |
|  |  |  |  |  | Job status/employment | Traumatic experiences |
|  |  |  |  |  | Language ability | Gender (being female) |
|  |  |  |  |  | Gender (being male) |  |
|  |  |  |  |  | Social relations |  |
| Taylor et al. - 2013 - Physical and Mental Health Status of Ira | PTSD, depression, & anxiety | Hopkins Symptom Checklist | Iraqi refugees in the US | 366 |  | Years of living in host country |
|  |  |  |  |  |  | Chronic health issues |
| Teodorescu et al. - 2012 - Mental health problems and post-migr | PTSD & Depression | Structured Clinical Interview for DSM-IV-TR PTSD Module | Refugees in Norway | 61 |  | Traumatic experiences |
|  |  |  |  |  |  | Postmigration stress |
|  |  |  |  |  |  | Unemployment |
| Tessitore, Parola, Margherita - 2022 - Mental Health Risk an | PTSD | Harvard Trauma Questionnaire | Nigerian male asylum seekers in Italy | 36 | Legal status | Postmigration stress |
|  |  |  |  |  |  | Gender norms and role expectations |
| Tinghög et al. - 2017 - Prevalence of mental ill health, trauma | PTSD | Harvard Trauma Questionnaire | Syrian refugees in Sweden | 1215 |  | Traumatic experiences |
|  | Depression & Anxiety | Hopkins Symptom Checklist |  |  |  | Discrimination |
|  |  |  |  |  |  | Language difficulty |
|  |  |  |  |  |  | Loneliness |
| Tippens et al-2021-Psychological Distress Prevalence | Psychological distress | Self-Reporting Questionnaire | Congolese adults in Kenya | 245 | Gender (being male) | Age (being older) |
|  |  |  |  |  | Religiosity | Loneliness |
|  |  |  |  |  | Marital status (being married) | Problems accessing services |
| Torres et al. - 2022 - Refugees in Brazil An investigation of S | Psychological distress | Psychophysical distress inventory | Syrian refugees in Brazil | 202 |  | Strong intra ethnic identity |
| Tufan, Alkin, Bosgelmez - 2013 - Post-traumatic stress disorder | PTSD | PTSD Checklist | Asylum seekers in Turkey | 57 | Social support | Traumatic experiences |
| Turner et al. - 2003 - Mental health of Kosovan Albanian refuge | PTSD | Post-traumatic Diagnostic Scale | Kosovan refugees in the UK | 842 |  | Age (being older) |
|  | Depression | Beck Depression Inventory |  |  |  | Traumatic experiences |
| Um et al. - 2015 - Correlates of depressive symptoms among Nort | Depression | Center for Epidemiologic Studies Depression Scale | North Korean refugees in South Korea | 304 | Integration/acculturation | Discrimination |
|  |  |  |  |  | Social relations |  |
|  |  |  |  |  | Good physical health |  |
| Uribe Guajardo et al. - 2016 - Psychological distress is influe | Depression & Anxiety | Kessler Psychological Distress Scale | Iraqi refugees who were granted permanent residence in Australia | 450 |  | Years of living in host country |
|  |  |  |  |  |  | Age (being older) |
| Van Heemstra et al. - 2020 - Contextualizing Cognitions the Rel | PTSD | Harvard Trauma Questionnaire | Palestinian refugees in West Bank, Palestine | 85 | Coping strategy | Traumatic experiences |
|  |  |  |  |  |  |  |
| Von Haumeder, Ghafoori, Retailleau - 2019 - Psychological adapt | PTSD | The PTSD Checklist | Syrian refugees in Germany | 127 | Self-efficacy | Unemployment |
|  |  |  |  |  |  | Lack of recognizing of previous knowledge and skill |
| Vonnahme et al. - 2015 - Factors Associated with Symptoms of De | Depression | Hopkins Symptom Checklist | Bhutanese refugees in the US | 386 | Social support | Financial strain |
|  |  |  |  |  |  | Experience of identity threat |
|  |  |  |  |  |  | Postmigration stress |
|  |  |  |  |  |  | Maladaptive coping |
| Vromans et al. - 2020 - Persistent psychological distress in | Depression & Anxiety | Hopkins Symptom Checklist | Female refugees from various countries in Australia | 83 |  | Traumatic experiences |
|  |  |  |  |  |  | Postmigration stress |
| Walther et al. - 2020 - Living Conditions and the Mental Health | Psychological distress | Patient Health Questionnaire for Depression and Anxiety | Refugees and asylum seekers from various countries who arrived in Germany | 4325 |  | Gender (being female) |
|  |  |  |  |  | Ethnic identity | Age (being older) |
|  |  |  |  |  | Living place | Marital status |
|  |  |  |  |  | Job status/employment | Traumatic experiences |
|  |  |  |  |  | Integration/acculturation | Postmigration stress |
|  |  |  |  |  | Years of living in host country |  |
| Walther et al. - 2020 - Psychological distress among refugee | Psychological distress | Refugee Health Screener | Refugees from various countries who arrived in Germany | 2639 | Integration/acculturation | Legal status |
|  |  |  |  |  |  | Living conditions |
|  |  |  |  |  |  | SES |
|  |  |  |  |  |  | Unemployment |
|  |  |  |  |  |  | Gender (being male) |
| Westermeyer, Neider, Vang - 1984 - Acculturation and mental hea | Depression | Zung Scale for Depression | Hmong refugees in the US | 97 |  | Chronic health issues |
|  |  |  |  |  |  | Chronic mental health issues |
|  |  |  |  |  |  | Language difficulty |
|  |  |  |  |  |  | Financial strain |
| Woltin et al. -2018 (1) - Regulatory focus, coping strategies, and | Depression & Anxiety | Hopkins Symptom Checklist | Syrian refugees in Turkey | 273 | Self-regulatory focus | Maladaptive coping |
| Woltin et al. -2018 (2) - Regulatory focus, coping strategies, and | Depression & Anxiety | Hopkins Symptom Checklist | Syrian refugees in Germany | 169 | Self-regulatory focus | Maladaptive coping |
| Yamin - 2021-The Development of Posttraumatic Stress Disorder | PTSD | PTSD Checklist | Iraqi refugees in the United States. | 53 | Acculturation | Chronic health issues |
|  | Depression | Hospital Anxiety and Depression Scale |  |  |  |  |
| Ying et al. - 1997 - Psychological dysfunction in Southeast Asi | Depression & Anxiety | Florida Health and Family Life Instrument | Southeast Asian refugees in the US | 2234 | Sense of coherence | Traumatic experiences |
|  |  |  |  |  | Education | Gender (being male) |
|  |  |  |  |  | Language ability |  |
| Yun et al. - 2021 - The Relationship Between Acculturative S | Depression & Anxiety | Hopkins Symptom Checklist | Iraqi refugees in the US | 219 |  | Postmigration stress |
|  |  |  |  |  |  |  |
| Yun, Kim - 2021 - Torture Experiences and Mental Health Problem | PTSD | Impact of Event Scale | North Korean refugees in South Korea | 642 | Social support | Traumatic experiences |
|  | Depression | Center for Epidemiological Study-Depression |  |  |  | Gender (being female) |
|  | Anxiety | Beck Anxiety Inventory |  |  |  | Chronic health issues |
