# Supplementary material for: The Socio-Ecological Factors Associated with Mental Health Problems and Resilience in Refugees: A Systematic Scoping Review
Source: Trauma Violence Abuse. 2024 Oct 8;26(3):598–616. doi: 10.1177/15248380241284594 (PMC12145474; doi:10.1177/15248380241284594)
Supplement: sj-docx-5-tva-10.1177_15248380241284594 – Supplemental material for The Socio-Ecological Factors Associated with Mental Health Problems and Resilience in Refugees: A Systematic Scoping Review [file sj-docx-5-tva-10.1177_15248380241284594.docx]

**Supplemental Material 5: Results Summary of Individual Qualitative Studies**

| **Authors** | **Outcomes** | **Outcome variables measurement** | **Samples** | **Sample Size** | **Protective factors** | **Risk Factors** |
| --- | --- | --- | --- | --- | --- | --- |
| Abraham-2018-Coping-resilience-and-posttraumatic | Resilience | Focus group discussion and in-depth interview. | Female Eritrean refugees aged 18–60 in Norway | 18 | Religiosity | Traumatic experiences |
|  |  |  |  |  | Social support | Time and experience in refugee camp |
|  |  |  |  |  | Social relations |  |
|  |  |  |  |  | Language ability |  |
|  |  |  |  |  | Gratitude |  |
|  |  |  |  |  | Hope and positive outlook |  |
| Abur, Mphande - 2020 - Mental Health and Wellbeing of South Sud | PTSD | A series of semi-structured, one-on-one interview | South Sudanese-Australians living in Melbourne ages ranging from 18 to 64 years | 20 | Exercise and being active (Emotion-focused) | Discrimination |
|  |  |  |  |  | Access to opportunities | Postmigration stress |
|  |  |  |  |  | Employment | Unemployment |
|  |  |  |  |  | Education | Financial strain |
|  |  |  |  |  |  | Family dysfunction |
|  |  |  |  |  |  | Social isolation |
|  |  |  |  |  |  | Chronic mental health issues |
| Affleck-2018-If-one-does-not-fulfil-his-duties-h | Resilience | Ethnographic observations, key informant interviews and shadowing, and qualitative interviews. | Sri Lankan Tamil refugee aged 20 to 60 in Canada | 33 | Structured daily activities (Emotion-focused) | Traumatic experiences |
|  |  |  |  |  | Mindfulness (Emotion-focused) | Postmigration stress |
|  |  |  |  |  |  | Lack of recognition of pre-existing qualifications |
|  |  |  |  |  |  | Gender norms and role expectations |
|  |  |  |  |  |  | Loss of status |
|  |  |  |  |  |  | Family dysfunction |
| Akinyemi, Owoaje, Cadmus - 2016 - In Their Own Words Mental Hea | Mental health | Focus group discussions (FGDs) | Liberian refugees from 18 to 67 years in Nigeria | 32 | Religiosity | Discrimination |
|  |  |  |  |  | Good physical health | Unemployment |
|  |  |  |  |  |  | Living conditions |
|  |  |  |  |  |  | Social vices |
|  |  |  |  |  |  | Family dysfunction |
| Alachkar - 2022 - The lived experiences of resilience among Syr | Resilience | Semi-structured interviews | Syrian refugees ranging from mid-20s to mid-40s in the UK | 8 | Social support |  |
|  |  |  |  |  | Sense of purpose |  |
|  |  |  |  |  | Religiosity |  |
|  |  |  |  |  | Hope and positive outlook |  |
|  |  |  |  |  | Structured daily activities |  |
| Alemi, James, Montgomery - 2016 - Contextualizing Afghan refuge | Depression | In-depth interviews | Afghans age 36 to 71 years in San Diego, USA | 18 |  | Traumatic experiences |
|  |  |  |  |  |  | Postmigration stress |
|  |  |  |  |  |  | Language difficulty |
|  |  |  |  |  |  | Financial strain |
|  |  |  |  |  |  | Lack of recognition of pre-existing qualifications |
|  |  |  |  |  |  | Living conditions |
|  |  |  |  |  |  | Loss of status |
|  |  |  |  |  |  | Experienced/feelings of identity threat |
| Atari-khan et al. - 2021 - Concepts of Resilience Among Trauma- | Resilience | In-depth semi-structured interviews | Syrian adults ages ranged from 27 to 50 in the United States. | 8 | Social support | Trauma experiences |
|  |  |  |  |  | Personality traits | Financial strain |
|  |  |  |  |  | Sense of purpose | Language difficulty |
|  |  |  |  |  | Religiosity | Chronic health issues |
|  |  |  |  |  |  | Postmigration stress |
| Aube-2019-La-maison-bleue-strengthening-resil | Resilience | Observation and interview. | Refugees from mixed countries in Canada | 24 | Religiosity | Traumatic experiences |
|  |  |  |  |  | Social relations | Discrimination |
|  |  |  |  |  | Language ability | Postmigration stress |
|  |  |  |  |  | Hope and positive outlook | Language difficulty |
|  |  |  |  |  | Sense of purpose | Dealing with complicated paperwork |
|  |  |  |  |  | Sense of safety | Financial strain |
|  |  |  |  |  | Maintaining cultural identity |  |
| Babatundesowole-2020-Resilience-of-african-migrant-women | Resilience | A semi-structured interview. | West African adult women refugees in Sydney | 22 | Religiosity | Traumatic experiences |
|  |  |  |  |  | Social relations | Discrimination |
|  |  |  |  |  | Being economically empowered | Time and experience in camp |
|  |  |  |  |  | Hope and positive outlook | Police/law problems |
|  |  |  |  |  | Contributing to new community | Experienced/feelings of identity threat |
|  |  |  |  |  | Personality traits |  |
| Bjertrup et al. - 2018 - A life in waiting Refugees’ mental hea | Anxiety | In-depth interviews and focus group discussions | Syrian refugees aged 18–40 years in Greece | 56 |  | Disruptive lives |
|  |  |  |  |  |  | Social isolation |
|  |  |  |  |  |  | Uncertainty |
|  |  |  |  |  |  | Chronic mental health issues |
| Cenat et al. -2020-Multiple Traumas Health Problems and Resilie | Resilience | Individual semi-structued interview. | Haitian asylum seekers aged between 22 and 58 in Canada | 16 | Social support | Traumatic experiences |
|  |  |  |  |  | Hope and positive outlook | Chronic mental health issues |
| Corley-2020-Exploring-african-immigrant-womens- | Resilience | Semi-structured interview and focus group discussion. | African adult refugee women in the US | 39 | Religiosity | Traumatic experiences |
|  |  |  |  |  | Social support | Discrimination |
|  |  |  |  |  | Being economically empowered | Within community related stressors |
|  |  |  |  |  | Artistic expression (Emotion-focused) |  |
|  |  |  |  |  | Exercise and being active (Emotion-focused) |  |
|  |  |  |  |  | Access to opportunities |  |
| Davis - 2000 - Refugee Experiences and Southeast Asian Women's | PTSD | Open-ended interviews | Vietnamese refugees aged 21 to 67 in the US | 19 | Social support |  |
|  |  |  |  |  | Social relations |  |
|  |  |  |  |  | Hope and positive outlook |  |
| Denzongpa-2020-We-can’t-step-back-women-speciallya- | Resilience | Observational field notes, formal and informal interview. | A Bhutanese refugee woman in the US | 46 | Contributing to new community | Chronic health issues |
|  |  |  |  |  | Personality traits |  |
| Dieterich-Hartwell -2021- Refugees and DMT | Resilience | Movement sessions, survey, and semi-structured interview. | Refugees from mixed origins in the US aged 20-69 years | 13 | Religiosity | Lack of recognition of pre-existing qualifications |
|  |  |  |  |  | Social support |  |
|  |  |  |  |  | Body knowledge and movement |  |
|  |  |  |  |  | Hope and positive outlook |  |
|  |  |  |  |  | Sense of purpose |  |
|  |  |  |  |  | Focus on growth and self-development |  |
|  |  |  |  |  | Acculturation |  |
| Dubus -2022- Resiliency with forced migrants | Resilience | Interviews | Refugees aged 22-88 years old from various countries | 34 | Self-agency |  |
|  |  |  |  |  | Digital support |  |
|  |  |  |  |  | Social support |  |
|  |  |  |  |  | Maintaining cultural identity |  |
| Flothmann -2021- Seeking asylum in Bristol | Resilience | Semi-structured interviews | Refugees age between 20 and 50 years from Africa, the Middle East and Central Asia in the UK | 9 | Social relations | Within-community related stressors |
|  |  |  |  |  | Social support |  |
|  |  |  |  |  | Religiosity |  |
| Ganassin & Young - 2020 - From surviving to thriving | Resilience | Individual semi-structured interview. | Refugees from Middle East and Africa aged 30-47 years in the UK | 5 | Social relations | Language difficulty |
|  |  |  |  |  | Self-agency | Financial strain |
|  |  |  |  |  | Language ability | Lack of recognition of pre-existing qualifications |
|  |  |  |  |  | Hope and positive outlook |  |
|  |  |  |  |  | Contributing to new community |  |
| Goodman-2017-Trauma-and-resilience-among-refugee | Resilience | Individual in-depth interview. | Refugee woman from South and Central America in the US aged 26-62 years | 19 | Religiosity | Traumatic experiences |
|  |  |  |  |  | Social support | Discrimination |
|  |  |  |  |  | Social relations | Unemployment |
|  |  |  |  |  | Cognitive reframing (Emotion-focused) | Lack of recognition of pre-existing qualifications |
|  |  |  |  |  | Access to opportunities | Chronic health issues |
|  |  |  |  |  |  | Dysfunctional coping |
|  |  |  |  |  |  |  |
| Groen et al. - 2019 - Cultural identity confusion and psychopat | PTSD | The Brief Cultural Interview | Afghan and Iraqi refugee in the Netherlands with mean age 36 years | 57 |  | Strong intra ethnic identity |
|  |  |  |  |  |  | Postmigration stress |
| Han et al. - 2020 - Depression in North Korean refugees a mixed | Depression | An in-depth qualitative interview | North Korean refugees in South Korea aged 37-74 | 11 | Social support | Traumatic experiences |
|  |  |  |  |  | Personality traits | Postmigration stress |
|  |  |  |  |  |  | Loneliness |
| Hasan, et al. - 2018 - Exploring the role of faith in | Resilience | Open-ended semi-structured interview questions | Syrian refugees in the US aged 18-50 years | 10 | Religiosity | Postmigration stress |
|  |  |  |  |  | Sense of safety | Language difficulty |
|  |  |  |  |  | Maintaining cultural identity | Unemployment |
|  |  |  |  |  | Confidence in self identity | Experienced/feelings of identity threat |
| Hormozi-2018-First-generation-iranian-refugees-a | Resilience | Interview | Iranian refugees aged 25-76 years in the US | 10 | Religiosity | Postmigration stress |
|  |  |  |  |  | Social relations |  |
|  |  |  |  |  | Language ability |  |
|  |  |  |  |  | Personality traits |  |
| Kuttikat et al. - 2018 - Battered but gold: Sri Lankan Tamil re | Resilience | In-depth, semi-structured interviews | Sri Lankan Tamil refugees aged 23-54 years in India | 15 | Religiosity | Traumatic experiences |
|  |  |  |  |  | Spirituality | Discrimination |
|  |  |  |  |  | Social support | Unemployment |
|  |  |  |  |  | Social relations | Financial strain |
|  |  |  |  |  | Hope and positive outlook | Time and experience in camp |
|  |  |  |  |  | Structured daily activities (Emotion-focused) |  |
|  |  |  |  |  | Access to opportunities |  |
|  |  |  |  |  | Maintaining cultural identity |  |
| Larios et al. - 2022 - Explanatory models of post-traumatic str | PTSD & Depression | Semi-structured focusgroup interviews based on vignettes | Afghan refugees between the ages of 18 and 47 in Norway | 27 | Social support | Postmigration stress |
|  |  |  |  |  | Religiosity | Financial strain |
|  |  |  |  |  | Set goals | Loneliness |
|  |  |  |  |  | Exercise & being active | Family conflict |
|  |  |  |  |  | Employment | Within-community related stressors |
| Lavie-ajayi-2016-A-qualitative-study-of-resilience-a | Resilience | In-depth, semi-structured group interviews | Asylum seekers from Darfur between the ages 27 and 38 in Israel | 8 | Social support | Traumatic experiences |
|  |  |  |  |  | Social relations |  |
|  |  |  |  |  | Hope and positive outlook |  |
|  |  |  |  |  | Focus on growth and self-development |  |
|  |  |  |  |  | Cognitive reframing (Emotion-focused) |  |
|  |  |  |  |  | Set goals (Problem-focused) |  |
|  |  |  |  |  | Contributing to new community |  |
|  |  |  |  |  | Confidence in self-identity |  |
|  |  |  |  |  | Employment |  |
| Lenette-2013-Everyday-resilience-narratives-of-s | Resilience | Participant observation, in-depth interview, and visual ethnography. | Single refugee women’s from Africa aged 30-50 years in Brisbane, Australia | 4 | Religiosity | Postmigration stress |
|  |  |  |  |  | Social support | Language difficulty |
|  |  |  |  |  | Self-agency | Within community related stressors |
|  |  |  |  |  | Gratitude |  |
|  |  |  |  |  | Sense of purpose |  |
|  |  |  |  |  | Maintaining cultural identity |  |
|  |  |  |  |  | Personality traits |  |
| Liu et al.-2020- Strengths-based inquiry of resiliency factors | Resilience | Semi-structured individual interviews. | Adult refugees from various countries in Canada | 21 | Religiosity |  |
|  |  |  |  |  | Social support |  |
|  |  |  |  |  | Hope and positive outlook |  |
|  |  |  |  |  | Cognitive reframing (Emotion-focused) |  |
|  |  |  |  |  | Set goals (Problem-focused) |  |
|  |  |  |  |  | Contributing to new community |  |
|  |  |  |  |  | Access to opportunities |  |
|  |  |  |  |  | Sense of safety |  |
|  |  |  |  |  | Maintaining cultural identity |  |
|  |  |  |  |  | Acculturation |  |
|  |  |  |  |  | Personality traits |  |
|  |  |  |  |  | Gender (being female) |  |
|  |  |  |  |  | Employment |  |
|  |  |  |  |  | Education |  |
| Lusk et al.- 2019- Resilience, faith, and social supports | Resilience | Semi-structured interview. | Refugees from Central America aged 18 to 49 years in the US | 30 | Religiosity |  |
|  |  |  |  |  | Sense of purpose |  |
|  |  |  |  |  | Personality traits |  |
| Maleku et al. -2022- Conceptualizing mental health through | Depression & Anxiety | Virtual FGD using a secure Zoom platform | Bhutanese refugees between 20-30 years of age in the US | 46 | Social support | Dysfunctional coping |
|  |  |  |  |  | Digital support |  |
|  |  |  |  |  | Exercise & being active |  |
|  |  |  |  |  | Mindfulness experience |  |
|  |  |  |  |  | Artistic expression |  |
|  |  |  |  |  | Religiosity |  |
| Maung et al. - 2021 - Burmese Refugee Women in the Midwest N | Resilience | Semi-structured interview | Female Burmese refugees ages ranged from 22 to 57 years in the US | 11 | Religiosity |  |
|  |  |  |  |  | Social support |  |
|  |  |  |  |  | Gratitude |  |
|  |  |  |  |  | Hope and positive outlook |  |
|  |  |  |  |  | Exercise and being active (Emotion-focused) |  |
|  |  |  |  |  | Cognitive reframing (Emotion-focused) |  |
|  |  |  |  |  | Personality traits |  |
| Melamed-2019-Social-resilience-and-mental-health | Resilience | In-depth interviews. | Asylum-seekers from Eritrea aged 20-35 years in Switzerland | 10 | Social relations | Traumatic experiences |
|  |  |  |  |  | Self-agency | Discrimination |
|  |  |  |  |  | Contributing to new community | Problems accessing services |
|  |  |  |  |  | Maintaining cultural identity |  |
|  |  |  |  |  | Personality traits |  |
| Miller et al. - 2002 - Bosnian refugees and the stressors of ex | PTSD | Semi-structured interviews | Adult Bosnian refugees in Chicago, USA | 28 |  | Postmigration stress |
|  |  |  |  |  |  | Language difficulty |
|  |  |  |  |  |  | Financial strain |
|  |  |  |  |  |  | Chronic health issues |
|  |  |  |  |  |  | Loss of status |
|  |  |  |  |  |  | Loneliness |
|  |  |  |  |  |  | Social isolation |
| Munt-2012-Journeys-of-resilience-the-emotiona | Resilience | Discussions  Exit interviews | Refugee women from various countries aged 24-65 years in the UK | 9 | Religiosity | Discrimination |
|  |  |  |  |  | Spirituality |  |
|  |  |  |  |  | Social relations |  |
|  |  |  |  |  | Exercise and being active (Emotion-focused) |  |
|  |  |  |  |  | Maintaining cultural identity |  |
| Muruthi et al. - 2020 - We Pray as a Family The Role of Reli | Resilience | Ethnographic methods and used in-depth semi-structured interviews, daily observation, and spent time with families in their homes and attended community events over the course of two years | Karen refugees between ages 18 years and 60 years in the US | 14 | Religiosity |  |
| Paloma-2020-A-peer-support-and-peer-mentoring-a | Resilience | Session observation, written evaluation, and field notes. | Refugees from various countries aged 20-64 years in Spain | 10 | Social support |  |
|  |  |  |  |  | Self-efficacy |  |
|  |  |  |  |  | Hope and positive outlook |  |
|  |  |  |  |  | Problem-focused |  |
|  |  |  |  |  | Contributing to new community |  |
| Pearce-2016-Searching-for-tomorrowsouth-sudanes | Resilience | Photovoice: a visual participatory research tool | South Sudanese refugee women age from 25-68 years in Canada | 8 | Religiosity |  |
|  |  |  |  |  | Spirituality |  |
|  |  |  |  |  | Social support |  |
| Pineteh - 2017 - Moments of suffering, pain , and resilience | Resilience | An in-depth semi-structured interview method. | 30 Somali refugees in Cape Town, South Africa between the ages of 25 and 45 | 30 | Self-agency | Discrimination |
|  |  |  |  |  | Cognitive reframing (Emotion-focused) |  |
|  |  |  |  |  | Confidence in self identity |  |
| Rahapsari & Hill-2019-The body against the tides: a pilot study | Resilience | Verbal interviews with movement elicitation (ME) | Burmese refugees aged 37-45 years in the US | 3 | Spirituality | Discrimination |
|  |  |  |  |  | Social support | Postmigration stress |
|  |  |  |  |  | Body knowledge and movement | Unemployment |
|  |  |  |  |  | Language ability | Financial strain |
|  |  |  |  |  | Being economically empowered | Unsafe environment |
|  |  |  |  |  | Set goals (Problem-focused) | Within community related stressors |
|  |  |  |  |  | Personality traits |  |
| Schweitzer, Greenslade, Kagee - 2007 - Coping and resilience in | Resilience | A semi-structured interview | Resettled Sudanese refugees aged between 17 and 44 years in Australia | 13 | Religiosity | Unemployment |
|  |  |  |  |  | Social support | Lack of recognition of pre-existing qualifications |
|  |  |  |  |  | Comparison with-others (Emotion-focused) | Within community related stressors |
|  |  |  |  |  | Personality traits |  |
| Simich, Este, Hamilton - 2010 - Meanings of home and mental wel | Resilience | In-depth qualitative interviews | Sudanese refugees in Canada aged 20-60  years old | 30 | Social support | Gender norms and role expectations |
|  |  |  |  |  | Self-agency | Family conflict |
|  |  |  |  |  | Contributing to new community | Lack of social support |
|  |  |  |  |  | Sense of safety |  |
|  |  |  |  |  | Maintaining cultural identity |  |
| Sundvall et al. - 2020 - Safe but isolated – an interview study | Depression | Semi-structured interviews,  a biographical network map, and three health assessment scales. | Iraqi refugees in Sweden aged 23-71 | 31 |  | Discrimination |
|  |  |  |  |  |  | Unemployment |
|  |  |  |  |  |  | Financial strain |
| Terrana et al. - 2022-Foundations of Somali Resilience | Resilience | Virtual focus group discussions | Somali refugees over the age of 18 in San Diego | 22 | Collective identity | Discrimination |
|  |  |  |  |  | Religiosity | Problems accessing services |
|  |  |  |  |  | Social support |  |
| Thomas-2011-Resilience-of-refugees-displaced-in | Resilience | Focus groups and semi-structured interviews were used supported by the use of the Photovoice methodology. | Pakistani and Somali refugees aged 23-47 years in Nepal | 24 | Religiosity | Traumatic experiences |
|  |  |  |  |  | Social support | Discrimination |
|  |  |  |  |  | Sense of purpose | Unemployment |
|  |  |  |  |  | Contributing to new community | Lack of recognition of pre-existing qualifications |
|  |  |  |  |  |  | Police/law problems |
| Tippens et al. - 2021 - Cultural Bereavement and Resilience in | Resilience | Photovoice | Yazidi Women who were 19 years or older in the United States | 9 | Maintaining cultural identity |  |
|  |  |  |  |  | Religiosity |  |
| Tippens-2016-Urban-congolese-refugees-in-kenya-t | Resilience | In-depth semi-structured interviews and ethnographic participant observation | Congolese Refugees aged 18-70 years in Kenya | 55 | Religiosity | Financial strain |
|  |  |  |  |  | Social support | Police/law problems |
|  |  |  |  |  | Cognitive reframing (Emotion-focused) |  |
|  |  |  |  |  | Maintaining cultural identity |  |
| Udwan et al. - 2020 - Digital resilience tactics of Syrian refu | Resilience | In-depth interviews | Syrian refugees aged 18-39 years in the Netherlands | 22 | Social support |  |
|  |  |  |  |  | Social relations |  |
|  |  |  |  |  | Digital support |  |
|  |  |  |  |  | Access to opportunities |  |
| Verreault - 2017 - Dance/movement therapy and resilience buildi | Resilience | Post-session observation notes, FGD, individual interviews. | Female asylum seekers and refugees from various countries aged 19-50 years in the Netherlands | 8 | Religiosity |  |
|  |  |  |  |  | Spirituality |  |
|  |  |  |  |  | Social support |  |
|  |  |  |  |  | Social relations |  |
|  |  |  |  |  | Self-agency |  |
|  |  |  |  |  | Body knowledge and movement |  |
|  |  |  |  |  | Gratitude |  |
|  |  |  |  |  | Mindfulness (Emotion-focused) |  |
|  |  |  |  |  | Sense of safety |  |
|  |  |  |  |  | Maintaining cultural identity |  |
| Waanzi Hillary, Braak - 2022 - When the world turns upside down | Resilience | Open-ended life history interviews and semi-structured interviews | Zande Refugees in Uganda | 5 | Cognitive reframing | Within-community related stressors |
|  |  |  |  |  | Exercise & being active |  |
|  |  |  |  |  | Religiosity |  |
|  |  |  |  |  | Sense of purpose |  |
|  |  |  |  |  | Social relations |  |
| Walther et al. - 2021- A qualitative study on resilience in | Resilience | Semi-structured interview | Refugees from Syria and Afghanistan aged 18-55 years in Germany. | 54 | Cognitive coping strategies |  |
|  |  |  |  |  | Personality traits |  |
|  |  |  |  |  | Contributing to new community |  |
|  |  |  |  |  | Social relations |  |
|  |  |  |  |  | Social support |  |
|  |  |  |  |  | Acculturation |  |
|  |  |  |  |  | Sense of safety |  |
|  |  |  |  |  | Sense of purpose |  |
|  |  |  |  |  | Age (being younger) |  |
| Welsh, Brodsky - 2010 - After every darkness is light Resilient | Resilience | Semi-structured interviews | Afghan refugees aged 20–73 in the US | 8 | Religiosity |  |
|  |  |  |  |  | Social support |  |
|  |  |  |  |  | Gratitude |  |
|  |  |  |  |  | Hope and positive outlook |  |
|  |  |  |  |  | Sense of purpose |  |
|  |  |  |  |  | Focus on growth and self-development |  |
|  |  |  |  |  | Problem-focused |  |
|  |  |  |  |  | Personality traits |  |
| Yotebieng et al. - 2018 - Is well-being possible | Resilience | Observations, unstructured interviews and group discussions | African refugees aged 22-73 years in Cameroon, Africa. | 81 | Social support | Traumatic experiences |
|  |  |  |  |  | Social relations | Discrimination |
|  |  |  |  |  | Self-agency | Postmigration stress |
|  |  |  |  |  | Cognitive reframing (Emotion-focused) | Financial strain |
|  |  |  |  |  |  | Unsafe environment |
|  |  |  |  |  |  | Chronic health issues |
|  |  |  |  |  |  | Legal status |
|  |  |  |  |  |  | Problems accessing services |
